# Shedding light on the *DICER1* mutational spectrum of uncertain significance in malignant neoplasms

**DOI:** 10.3389/fmolb.2024.1441180

**Published:** 2024-10-03

**Authors:** D. S. Bug, I. S. Moiseev, Yu. B. Porozov, N. V. Petukhova

**Affiliations:** ^1^ Bioinformatics Research Center, Pavlov First Saint Petersburg Medical State University, St. Petersburg, Russia; ^2^ R. M. Gorbacheva Scientific Research Institute of Pediatric Hematology and Transplantation, Pavlov First Saint Petersburg State Medical University, St. Petersburg, Russia; ^3^ St. Petersburg School of Physics, Mathematics, and Computer Science, HSE University, Saint Petersburg, Russia; ^4^ Advitam Laboratory, Belgrade, Serbia

**Keywords:** Dicer1, variant of uncertain significance, variant effect prediction, gene evolution, oncology, molecular dynamics

## Abstract

The Dicer protein is an indispensable player in such fundamental cell pathways as miRNA biogenesis and regulation of protein expression in a cell. Most recently, both germline and somatic mutations in *DICER1* have been identified in diverse types of cancers, which suggests Dicer mutations can lead to cancer progression. In addition to well-known hotspot mutations in RNAase III domains, *DICER1* is characterized by a wide spectrum of variants in all the functional domains; most are of uncertain significance and unstated clinical effects. Moreover, various new somatic *DICER1* mutations continuously appear in cancer genome sequencing. The latest contemporary methods of variant effect prediction utilize machine learning algorithms on bulk data, yielding suboptimal correlation with biological data. Consequently, such analysis should be conducted based on the functional and structural characteristics of each protein, using a well-grounded targeted dataset rather than relying on large amounts of unsupervised data. Domains are the functional and evolutionary units of a protein; the analysis of the whole protein should be based on separate and independent examinations of each domain by their evolutionary reconstruction. Dicer represents a hallmark example of a multidomain protein, and we confirmed the phylogenetic multidomain approach being beneficial for the clinical effect prediction of Dicer variants. Because Dicer was suggested to have a putative role in hematological malignancies, we examined variants of *DICER1* occurring outside the well-known hotspots of the RNase III domain in this type of cancer using phylogenetic reconstruction of individual domain history. Examined substitutions might disrupt the Dicer function, which was demonstrated by molecular dynamic simulation, where distinct structural alterations were observed for each mutation. Our approach can be utilized to study other multidomain proteins and to improve clinical effect evaluation.

## 1 Introduction

Dicer1 is a double-stranded RNA (dsRNA) endoribonuclease playing a central role in short dsRNA-mediated post-transcriptional gene splicing. It is responsible for cleaving naturally occurring long dsRNAs and short hairpin pre-microRNAs (miRNA) into 21–23-nucleotide-long fragments with a two-nucleotide 3′ overhang, producing short interfering RNAs (siRNA) and mature microRNAs (miRNAs) (Ha and Kim, 2014; Yang and Lai, 2011; Foulkes et al., 2014). These small RNAs serve as guides that direct the RNA-induced silencing complex (RISC) to complementary RNAs for its degradation or translation prevention. Gene silencing mediated by siRNAs (RNA interference) controls the degradation of exogenous RNA along with the elimination of transcripts from mobile and repetitive DNA elements triggered by endogenous loci that affect gene expression and genome organization (Wilson and Doudna, 2013; Okamura and Lai, 2008). Thus, Dicer1 plays a key role in the overall protein translational control within the canonical miRNA biogenesis pathway (Fabian and Sonenberg, 2012).

Advances in understanding the genetic and molecular functions of Dicer1 have led to new insights into its role in cancer progression (Robertson et al., 2018; Caroleo et al., 2021; Vedanayagam et al., 2019). Mutations in the *DICER1* gene were associated with a predisposition to multiple cancer types—the *DICER1* syndrome—which is characterized by disrupted miRNA biogenesis and processing with subsequent disruption in the control of gene expression (Hill et al., 2009). Missense mutations associated with DICER1 syndrome were reported in various types of tumors: endocrine tumors, pleuropulmonary blastoma, cystic nephroma, rhabdomyosarcoma, multinodular goiter, thyroid cancer, ovarian Sertoli–Leydig cell tumor, neuroblastoma, and other neoplasias (Robertson et al., 2018). More than four thousand *DICER1* variants are available in the ClinVar database, which makes it the 19th most frequently mutated gene according to this database. Nearly half of the reported variants (2140) have unknown clinical effects, and the overwhelming majority of these are represented by missense mutations (Vogelstein et al., 2013).

Recent studies highlight the significance of miRNA biogenesis genes in hematological malignancies that are under mutational pressure during tumor progression. In particular, the downregulated expression of *DICER1* was revealed in mesenchymal stem cells (MSCs) from myelodysplastic syndrome patients (Santamaría et al., 2012). Furthermore, selective deletion of the *DICER1* gene in murine mesenchymal osteoprogenitors induces markedly disordered hematopoiesis with several MDS features, indicating the crucial role of this gene in mesenchymal “stroma” as a primary regulator of tissue function (Raaijmakers et al., 2010). Recent analysis of MDS clinical data revealed the high mutational burden in both miRNA processing genes and their association with common MDS mutations (Moiseev et al., 2021). Therefore, functional classification of variants that are currently listed as variants of uncertain significance is critically important for a fundamental understanding of *DICER1* functions as well as its role in cancer and utility in clinical diagnostics.

In this study, we evaluated the evolutionary history of Dicer1 and presented a multiple sequence alignment of Dicer1 orthologs suitable for the interpretation of variants observed in this gene. We also show that some evolutionarily intolerable variants negatively affect the structural stability of Dicer1.

## 2 Materials and methods

### 2.1 Homology study

We carried out a BLAST search of the human Dicer protein (isoform 1, accession number NP_001258211.1) against the NCBI RefSeq protein database (Altschul et al., 1990; O’Leary et al., 2016). The resulting hits were sorted by E-value, and the first 1,387 sequences, consisting of Dicer1 proteins, a known outgroup—insect Dicer2, and a number of similar proteins were aligned using the MAFFT algorithm v7 (Katoh et al., 2002). The maximum-likelihood tree was inferred from the acquired multiple sequence alignment (MSA) using iqTree utility v2 (Minh et al., 2020) with the LG + R10 model resolved by ModelFinder (Kalyaanamoorthy et al., 2017). Branch support was assessed with ultrafast bootstrap approximation [UFBoot (Minh et al., 2013; Hoang et al., 2017), 1,000 replicates]. We selected Dicer1 proteins from the tree, omitting Dicer2 paralogs, and generated a full-sequence MSA using MAFFT. Sequences with ambiguous amino acids were removed from the MSA, and misaligned amino acids were masked manually by observing the proximities of insertions and deletions in aligned sequences.

### 2.2 MSA refinement

Domain coordinates were obtained from PROSITE (Table 1) (Sigrist et al., 2013). Based on these coordinates, Dicer1 MSA was split into MSAs of its domains and non-domain subsequences, including interdomain, initial, and terminal sections that do not belong to any domain. All 15 subsequent MSAs were realigned by MAFFT, and then erroneous and incomplete sequences were discarded. Finally, the full-length Dicer1 MSA was assembled.

**TABLE 1:** Dicer domains.

Name (annotation rule)	Start	End
Helicase ATP-binding domain (PRU00541)	51	227
Helicase C-terminal domain (PRU00542)	433	602
Dicer double-stranded RNA-binding fold domain (PRU00657)	630	722
PAZ (PRU00142)	891	1,042
RNase III (PRU00177)	1,276	1,403
RNase III (PRU00177)	1,666	1,824
Double-stranded RNA-binding domain (PRU00266)	1,849	1,914

### 2.3 Selection of mutations for analysis

The missense mutations of *DICER1* in hematological malignancies were obtained from the COSMIC database (https://cancer.sanger.ac.uk/cosmic) (Tate et al., 2018) by filtering the variants in hematological and lymphoid tissues. Variants located in Dicer1 domains but not in RNase III were analyzed.

### 2.4 Protein structure modeling

All stages of protein modeling and analytical calculations were performed using the Schrödinger molecular modeling suite (version 2021-1) (Schrödinger, LLC, New York, NY, 2021). A Dicer full-length 3D-structure PDB ID AF-Q9UPY3-F1 predicted by AlphaFold (Jumper et al., 2021) was selected from the UniProt database (UniProt IDs Q9UPY3) (https://www.uniprot.org/). To ensure the AlphaFold structure was reliable and accurate, we performed the topological similarity analysis by TM-score calculation (Xu and Zhang, 2010) with the experimental Dicer structure: the TM-score was 0.8053 compared with 5ZAK for the Dicer model (Liu et al., 2018). The quality of the Dicer structure was tested and preprocessed in the Protein Preparation Wizard (PPW) (Madhavi Sastry et al., 2013). Detected problems and additional loop refinement were resolved in the Prime package (Jacobson et al., 2004). No problems were reported in the processed protein structure.

### 2.5 Molecular dynamics (MD) simulations

MD simulations were performed using the Desmond package (Bowers et al., 2006). The MD system was set up in “System Builder” in Maestro as follows: the TIP3P water model (Jorgensen et al., 1983) was used to simulate water molecules; the buffer distance in the orthorhombic box was set at 10 Å; a recalculated amount of Na+/Cl-ions were added to balance the system charge and placed randomly to neutralize the solvated system; additional salt was appended for final concentration 0.15 M in order to simulate physiological conditions.

Molecular dynamic simulations were conducted with the periodic boundary conditions in the NPT ensemble class using OPLS3e force field parameters (Harder et al., 2015; Roos et al., 2019). The temperature and pressure were kept at 300 K and 1 atmospheric pressure, respectively, using Nosé–Hoover temperature coupling and isotropic scaling (Nosé, 1984). The model system was relaxed before simulations using Maestro’s default relaxation protocol, which includes two stages of minimization (restrained and unrestrained), followed by four stages of MD runs with gradually diminishing restraints. MD simulations were carried out with 100 ns and 300 ns runs and recording the trajectory configurations obtained at 50 ps intervals.

### 2.6 Protein site-specific mutagenesis

Initially, the preprocessed and refined structure of wild-type Dicer was relaxed by MD simulation for 100 ns in order to obtain the relaxed system with minimized energy. The recorded trajectories were clustered, and the total energies of the representative structures were calculated in Prime (selected parameters VSGV and OPLS3e). The structure with the lowest energy was employed in further long MD simulations and protein mutagenesis. Specific mutations were introduced into the structure by the 3D Builder Panel in Maestro, and side-chain rotamers were refined. The local structure around the inserted mutation was minimized; the 10 amino acids loop around the introduced mutation was refined in the Prime package, followed by side-chain prediction to locate an appropriate residue conformation. The quality of the mutated model was validated in PPW as previously (Section 2.4), and given Dicer, mutated structures were subjected to 300-ns MD simulation.

### 2.7 Analysis of the MD simulation

The MD trajectory files were investigated by using simulation quality analysis (SQA) and simulation event analysis (SEA) along with simulation interaction diagram (SID) programs available with the Desmond module: root-mean-square deviation (RMSD), root-mean-square fluctuation (RMSF), total intra-molecular hydrogen bonds (Hbonds Intra), radius of gyration (Rgyr), and secondary structure elements (SSE) were calculated and visualized. The recorded trajectories were clustered, and the total energies of the representative structures were calculated in Prime (options VSGV and OPLS3e). Additionally, the H-bonds formed by mutated residue with the whole protein molecule were computed by analyse_trajectory_ppi.py script and SEA, and the interactions were compared with the WT structure. To characterize the local changes induced by mutation, the region radius of 10 Å around the introduced residue was analyzed by calculating local RMSD, H-bonds, Rgyr, and surface area (the clustered structures with minimal total energy were used to measure the surface area in 10 Å radius of mutated residue).

## 3 Results

### 3.1 Obtaining variants of uncertain significance in *DICER1*

**FIGURE 1:**
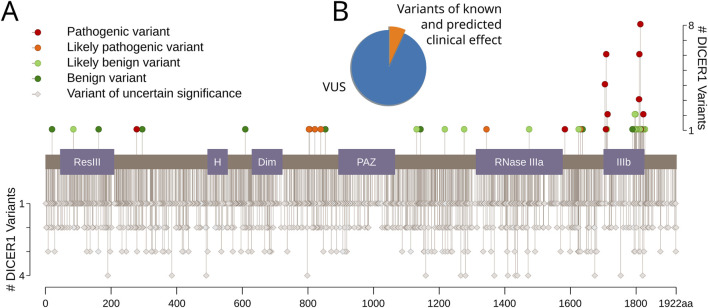
Dicer1 variants with known and predicted clinical effects. **(A)** The lolliplot of Dicer1 variants with different clinical effects from ClinVar (accessed December 2021). Protein domains are indicated as follows: ResIII: type III restriction enzyme, res subunit; H: Helicase conserved C-terminal domain; Dim: Dicer dimerization domain; PAZ: PAZ domain; RNase IIIa: ribonuclease IIIa domain; IIIb: ribonuclease IIIb domain. **(B)** Pie chart showing the distribution of variants with known and predicted clinical effects versus VUS.

We examined ClinVar (Landrum et al., 2017), a public archive of human genetic variants, to identify known and predicted pathogenic and benign amino acid substitutions in *DICER1*, as well as missense variants of uncertain significance (VUSs). In total, we found 2002 variants, and more than 91% of them are VUS (accessed March 2022). We found 45 variants annotated as intolerant (11 likely pathogenic and 34 pathogenic) and 44 variants annotated as tolerant (36 likely benign and eight benign) (Figure 1). Importantly, only six hotspot positions in Dicer1 have been reported: E1705, D1709, D1713, G1809, D1810, and E1813 (Chen et al., 2018; Klein et al., 2014).

VUSs present a substantial challenge in the clinical context (Federici and Soddu, 2020), and current efforts by the scientific community focus on developing easily applicable methods for their classification. Widely used variant effect prediction tools (CADD, PROVEAN, SIFT, PolyPhen, SNAP, PhD-SNP, and MAPP) were applied to identify missense mutations that are assumed to lead to *DICER1*-associated cancers. Surprisingly, one of the latest prediction models, EVE, did not provide resolution for mutations past position 1789, which leaves unresolved substitutions at several known hotspots, such as G1809, D1810, and E1813 (Kock et al., 2019). As for other tools, even in cases of known pathogenic mutations, their expected accuracy levels ranged from 65% to 80% (Thusberg et al., 2011). This low accuracy is primarily due to misalignments and the inclusion of low-quality sequences, paralogs, and remote homologs that are not functionally equivalent.

To overcome problems associated with the use of automated variant predictors, we constructed our own datasets of well-defined Dicer1 orthologs based on its evolutionary history and domain architecture and used these datasets to derive a risk map for *DICER1*-associated cancer.

### 3.2 Constructing the dataset

After sponges diverged from the main animal branch, but before the cnidarian split, *DICER1* was duplicated, resulting in two paralogs, *DICER1* and *DICER2*, (Mukherjee et al., 2012). The roles of these paralogs are different. Dicer1 functions in miRNA-based gene regulation (Welker et al., 2011), whereas Dicer2 is responsible for antiviral immunity (Kolaczkowski et al., 2010). As Dicer2 was subsequently lost in some metazoans, including vertebrates, Dicer1 gained some of its functions (Hammond, 2005). For clarification purposes, we will use the asterisk to label such a multifunctional DICER1* gene and its Dicer1* protein where necessary.

**FIGURE 2:**
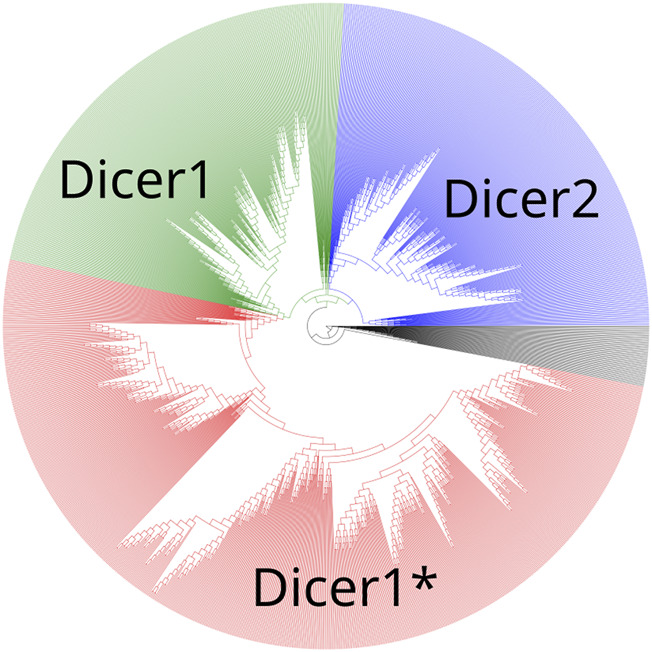
A maximum-likelihood phylogenetic tree of the Dicer group. Dicer1, Dicer2, and Dicer1* subclades are demonstrated.

To establish the precise evolutionary history of Dicer, we first collected its homologs by carrying out a BLAST search initiated with the human Dicer protein (isoform 1, accession number NP_001258211.1) against the NCBI RefSeq protein database (Altschul et al., 1990; O’Leary et al., 2016). The resulting hits were sorted by E-value, and the first 1,387 sequences, consisting of Dicer1 proteins, a known outgroup—insect Dicer2, and a number of similar proteins were aligned using the MAFFT algorithm v7 (Katoh et al., 2002). The maximum-likelihood tree was inferred from the acquired MSA using iqTree utility v2 (Minh et al., 2020) (Figure 2, Supplementary File S1) with the LG + R10 model resolved by ModelFinder (Kalyaanamoorthy et al., 2017). Branch support was assessed with ultrafast bootstrap approximation [UFBoot (Minh et al., 2013; Hoang et al., 2017), 1,000 replicates].

A maximum-likelihood phylogenetic tree showed two distinct clades corresponding to Dicer1 and Dicer2, and all Dicer1* sequences formed a distinct subclade within the Dicer1 group (Figure 2).

**FIGURE 3:**
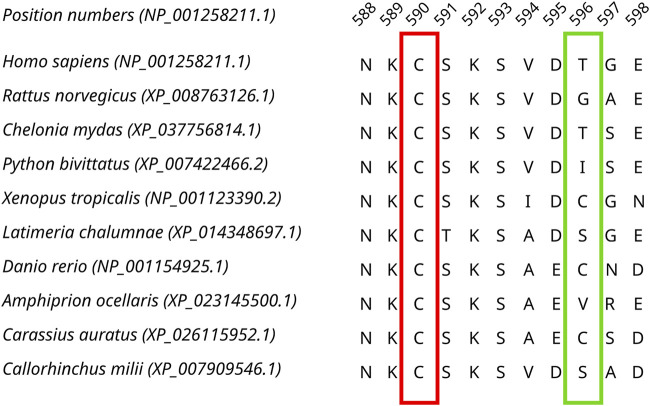
A fragment of the Dicer1* MSA corresponding to human Dicer1 sequence positions 588–598 demonstrating both conserved (outlined by the red rectangle) and variable (outlined by the green rectangle) positions in the dataset.

Sequences from the Dicer1* sub-clade were aligned, and by identifying both invariant and highly variable positions in the MSA (Figure 3), we concluded that there was enough time for orthologs to diverge.

Next, we inspected the alignment and noticed misalignments in some Dicer1 domains. To mitigate this problem, we split the MSA of full-length protein sequences into subsequences corresponding to human Dicer1 domain coordinates and realigned sequences of each domain separately. Erroneous and incomplete sequences were removed from domain MSAs. After the realignment, we reassembled the full-length Dicer1 MSA (termed “final MSA”), resulting in a reduction in the number of misaligned regions and improving predictions according to known clinical effects for some positions (Table 2).

**TABLE 2:** Examples of variant assessment before and after domain realignment.

Variant	Known clinical effect	Tolerant substitutions	
		Before the realignment	After the realignment
E1705K	Pathogenic	E, K, N	E
E1813K	Pathogenic	E, K, L, V	E
D1822V	Pathogenic	D, C, V, K, Y	D

### 3.3 Variant effect interpretation

**FIGURE 4:**
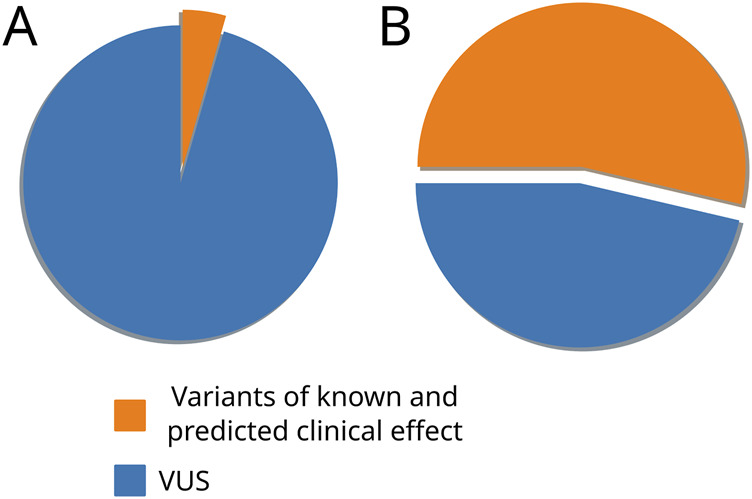
Pie charts representing the distribution of variants with known and predicted clinical effects and VUS before **(A)** and after **(B)** the phylogeny-based variant effect prediction.

We collected a total of 1834 unique missense VUSs from the ClinVar database, and their positions were examined in the final MSA. We adopted the following straightforward reasoning to evaluate variants, similar to a previously reported protocol (Adebali et al., 2016): if a variant occurs in an invariant position or if it is not seen in a highly conserved position of the final MSA, then it is intolerant. If a variant exists in any of the final MSA sequences, then it is evolutionarily allowable or tolerant. We also ensured that only single substitutions serve as evidence for benignity, and if each substitution in an examined position is accompanied by another one in an adjoining position, then the tested variant is uninterpretable. This approach allowed us to assign 485 variants as tolerant and 588 variants as intolerant and thus potentially damaging substitutions (Figure 4).

We also used the SAVER algorithm (Adebali et al., 2016) to evaluate variants against the final MSA, and it confirmed 1,067 of our 1,073 predictions. Satisfactorily, known *DICER1* hotspot mutations were evolutionarily intolerable in our final MSA and consequently were predicted as damaging (Table 2), thus providing a positive control for our analysis (Supplementary File S2).

Producing a high-quality final MSA of Dicer1 orthologs distinguishes our approach from automated variant predicting bioinformatics tools. For example, in our final MSA, position M1808 is invariable; therefore, any variant in this position is evolutionarily intolerant and thus damaging. It is worth noting that M1808 is adjacent to three known Dicer1 hotspots, G1809, D1810, and E1813, which further reinforces its potential significance. However, automated tools provide conflicting and erroneous assignments for a documented VUS in this position: M1808L (dbSNP ID: rs763241498), is predicted to be “possibly damaging” by PolyPhen2 (which is a less confident prediction than “probably damaging”), “tolerated” by SIFT, and “neutral” by PROVEAN, whereas EVE did not provide any interpretation of this variant. These erroneous assignments result from “noisy” MSAs used by these tools. For example, we have identified several paralogs (Dicer2 sequences) in some of these MSAs (Supplementary Figure S1).

### 3.4 Assessment of selected *DICER1* mutations in hematological malignancies

Advances in understanding the genetic and molecular functions of Dicer1 have opened new horizons into its role in cancer progression with questions that remain unanswered (Robertson et al., 2018; Caroleo et al., 2021). We made sure all known Dicer1 hotspots were completely conserved in the MSA and turned to less-studied cases. Recent studies highlight the significance of miRNA biogenesis genes in hematological malignancies that are under mutational pressure during tumor progression, and their disruption can alter the cellular proliferation through miRNA regulation. Therefore, the investigation of mutations’ pathogenicity in the context of oncohematology might shed light on the functional importance of these proteins and the mutations acquired under tumor evolution.

To demonstrate the validity of our approach, we selected four VUS that are located within functional domains of Dicer1 but outside known hot spots: Y124H (COSMIC database identificatory: COSV100601713), located in the Helicase ATP-binding domain, I445S (COSV58619533) and F508C (COSV58616328), located in the Helicase domain C-terminus), and T993R (COSV58617548), located in the PAZ domain. In addition to assessing the evolutionary tolerability of each variant, we performed molecular dynamics (MD) simulations of mutated Dicer1 proteins to evaluate potential structural alterations caused by these mutations.

**FIGURE 5:**
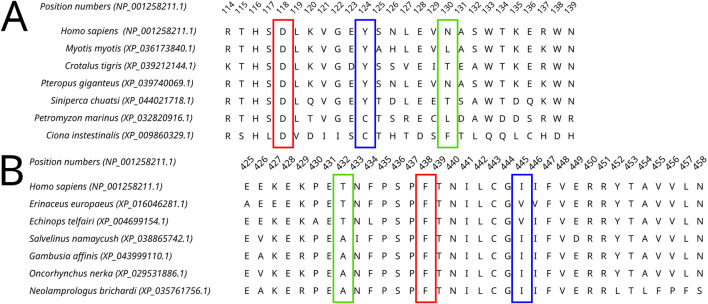
A fragment of the Dicer1* MSA corresponding to the human Dicer1 sequence around positions 124 **(A)** and 445 **(B)**, demonstrating the variability of these positions in the dataset.

All four selected variants were found to be evolutionarily intolerable by our approach. None of these specific substitutions were found in the multiple alignments of (i) Dicer1 orthologs that emerged after the last duplication event, leading to Dicer subfunctionalization (MSA1) or (ii) all identified Dicer orthologs (MSA2) (Figure 2). Two positions, corresponding to Y124 and I445, were variable. In MSA1, a single substitution in position 124 was found—Y124C in the Dicer1 sequence from *Petromyzon marinus* (Figure 5); however, no instances of Y124H were detected in either MSA. Thus, we interpret this variant as evolutionarily intolerable. Similarly, several instances of I445V substitution were detected in MSA1 (Figure 5), but there were no instances of I445S substitutions in either MSA. Consequently, this variant was also considered evolutionarily intolerant. The other two positions, F508 and T993, were invariable; therefore, reported VUSs F508C and T993R are evolutionarily intolerable (Supplementary Table S1).

MD simulations showed relative stability of all four mutated Dicer proteins compared to the wild-type protein (Supplementary Figure S2; Supplementary Table S2). The variants Y124 and I445S did not show significant bond alterations, which was demonstrated by the relative stability of structural elements during MD simulation (Supplementary Figures S3,S4).

**FIGURE 6:**
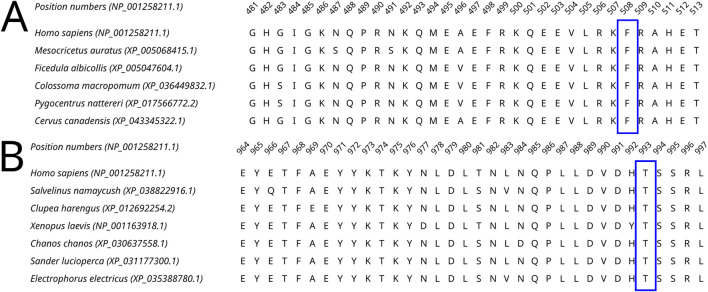
A fragment of the Dicer1* MSA corresponding to the human Dicer1 sequence around positions 508 **(A)** and 993 **(B)**, demonstrating the conservation of these positions in the dataset.

**FIGURE 7:**
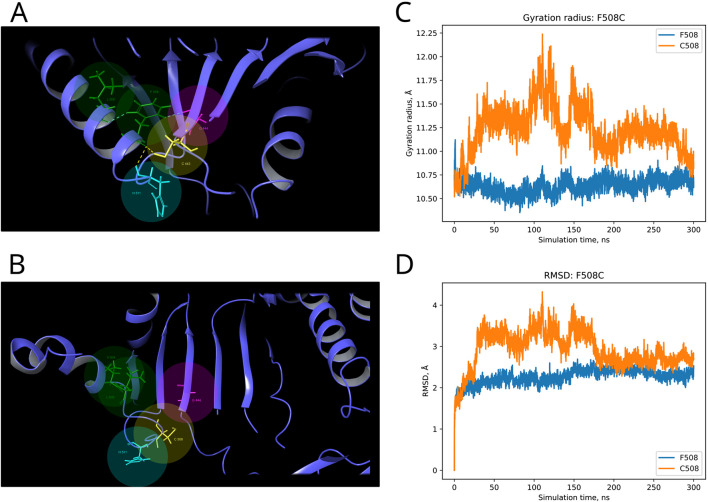
Structural alterations of Dicer1 variant F508C. **(A)** Interactions formed by wild-type amino acid F508. **(B)** Interactions formed by mutation C508. Amino acids taking part in bond formation are marked by spheres. H-bonds are indicated by dashed yellow lines, and aromatic H-bonds are indicated by dashed blue lines. Protein secondary structural elements (α-helixes, β-strands, and disordered loops) are shown in blue by cartoon representation. The radius of gyration **(C)** and RMSD **(D)** fluctuations of the 10Å region around the wild-type amino acid and corresponding mutation through a 300-ns MD simulation.

**FIGURE 8:**
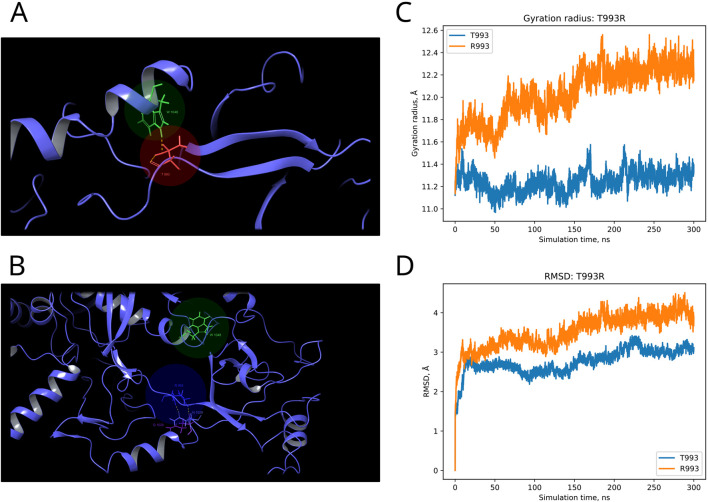
Structural alterations of Dicer1 variant T993R. **(A)** Interactions formed by wild-type amino acid T993. **(B)** Interactions formed by mutation R993. Amino acids taking part in bond formation are marked by spheres. H-bonds are indicated by dashed yellow lines, and aromatic H-bonds are indicated by dashed blue lines. Protein secondary structural elements (α-helixes, β-strands, and disordered loops) are shown in blue by cartoon representation. The radius of gyration **(C)** and RMSD **(D)** fluctuations of the 10Å region around the wild-type amino acid and corresponding mutation through a 300-ns MD simulation.

F and T are strongly conserved in the 508th and 993rd positions, respectively, by analyzed MSA, and other substitutions are evidently prohibited by evolution (Figure 6). These positions are also invariable in the majority of Dicer1* sequences, which underscores the importance of their conservation for the functionality of Dicer1 homologs in general. Neither F508C nor T993R is ever seen among Dicer1 homologous sequences, including Dicer2. The detailed damaging effect of these Dicer variants was confirmed by MD simulation. RMSD fluctuations of F508C and T993R are roughly 30% higher than wild-type protein, in particular for T993R, which triggers a more destabilized area; both the F508 and T993R regions are characterized by a significantly increased radius of gyration, indicating the loss of local compactness and more pronounced conformational changes (Figures 7, 8; Supplementary Table S3). Moreover, significant bond alterations were observed for F508 and T993R variants (Supplementary Figure S3). In particular, both induce the loss of five H-bonds within the considered 10Å region. The spectrum of the most frequent interactions of F508 consists of H-bonds with V504, H511, C443, G444, and L505 that are responsible for α-helix and β-sheet interposition. All these interactions were completely lost for C508, and the set of occurring bonds through the MD run was totally different. The differences led to severe structural changes: the α-helix containing residue 508 was partially disbanded along with loss of interactions with β-sheet; the whole local region was deformed with lower inter-compactness (Figure 7). Similar severe structural changes were characterized for T993R substitution: T993 and R993 have only R944 as a one-H-bond donor in common; therefore, the most frequent and stable interactions of T993 with W1048 and H856 were lost for the R993 mutant. Such a loss of an essential H-bond with W1048 leads to a significant distance increase between the corresponding α-helix and β-sheet, entire region deformation, and destabilization (Figure 8).

## 4 Discussion

The *DICER1* gene and its mutations draw interest from the carcinogenesis perspective as a crucial and irreplaceable player in miRNA and the siRNA biogenesis gene, while cancer pathogenesis is widely characterized by the dysfunction of the miRNA spectrum (Vedanayagam et al., 2019; Foulkes et al., 2014). Indeed, both germline and somatic mutations in DICER1 were identified in diverse types of cancer (Hill et al., 2009; Witkowski et al., 2013; Seki et al., 2014; Wu et al., 2018; Chen et al., 2015). We have analyzed *DICER1* variants available in the ClinVar database and found that 91% of registered variants are of unknown clinical significance. Among them, only six cancer-associated Dicer1 hotspots have been reported previously (Vedanayagam et al., 2019). In this case, the classification of the majority of *DICER1* variants and prediction of their clinical effects would benefit the comprehension of the *DICER1* role in tumorigenesis.

We applied widely used bioinformatic tools to evaluate the clinical effects of the mutations (CADD, PROVEAN, SIFT, PolyPhen, SNAP, PhD-SNP, and MAPP): unfortunately, the expected accuracy for even well-known *DICER1* hotspot mutations did not exceed 60%–80%. After applying a comparative genomic approach, these tools produced several issues and incorrect predictions, which are basically the result of faulty MSA. Most of the errors occur from the inclusion of low-quality sequences and paralogs in the analytic dataset. Therefore, we advocate for precise and individual dataset construction for each protein of interest based on its evolutionary history and domain architecture. For this purpose, we reconstructed *DICER1* evolution and divided two paralogs, Dicer1 and Dicer2, which, in addition to their sequence homology, are functionally different proteins (Welker et al., 2011; Kolaczkowski et al., 2010). Moreover, the last major evolutionary event in the history of *DICER1* homologs was the loss of *DICER2* (Mukherjee et al., 2012), and it is essential to take only Dicer1 sequences from proteomes without Dicer2. We inspected and refined the final MSA for the interpretation of Dicer1 variants. First, the MSA dataset was validated on the well-known protein hotspots. Our “straightforward” prediction approach was based on the total conservation of the position of interest and its neighboring positions corresponding to the human Dicer1 sequence, which means intolerance for substitution. If the position is changed along with its neighbors, we consider such situations as uncertain because the change of local context could compensate for the impact of the substitution on the functionality of the whole protein and, furthermore, on clinical significance. Thus, our approach allowed us to determine the potential significance of 1,073 variants: among them, 485 were tolerant, and 588 were intolerant. In addition, we thoroughly analyzed those variants whose predictions were not consistent with the automated tools’ predictions. Several pieces of evidence were demonstrated for such conflicting variants (e.g., M1808L), which are close to several well-known “hotspots.” This example clearly explains the issues in MSA of automated programs and consequent false predictions.

Moreover, our obtained MSA was applied for analysis of those *DICER1* variants that occurred in cancer where the role of this gene is of particular interest. Recent studies showed the potential *DICER1* involvement in hematological malignancies (Santamaría et al., 2012; Raaijmakers et al., 2010; Moiseev et al., 2021). Therefore, the variants with unknown significance were analyzed using our method in order to evaluate the potential effect on cancer progression. Dicer1 missense mutations that occurred in functional domains (Y124H (Helicase ATP-binding), I445S and F508C (Helicase C-terminal), and T993R (PAZ)) were analyzed by MSA. The assessment by comparative genomics was additionally compared with the evaluation of these variants by *in silico* site-specific mutagenesis and molecular dynamics simulation. In particular, the analysis of variants Y124H and I445S (both in the Helicase domain) demonstrated some variability of these protein positions compared to F508C (Helicase C-terminal) and T993R (PAZ), which were strongly conserved, and other substitutions are evidently prohibited by evolution. The results obtained by the MSA analysis were in compliance with those of the molecular dynamics simulation, which showed the structural consequences of mutations: namely, significant structural alterations in the Dicer1 mutated with F508C and T993R substitutions. In these cases, the key interactions were lost, which led to protein local region destabilization. F508C dramatically altered the mutual proximity of secondary structural elements within the C-terminal Helicase domain; T993R disrupted the interactions of the PAZ domain with both interdomain regions that, in turn, affect PAZ positioning between adjacent domains (Dicer dsRNA-binding fold and RNAase III). All these events are the distinct basis for protein dysfunction and/or dysregulation.

To summarize, in addition to the well-known *DICER1* tumor predisposition syndrome (González et al., 2021), the potential oncogenic role of this gene is being studied and discussed in other malignant diseases (Robertson et al., 2018). Our work was dedicated to investigating and clarifying the effect of the mutational spectrum across the whole protein sequence and marked as uncertain significance on the basis of the combination of in-depth gene evolution reconstruction and molecular modeling of mutational structural–functional consequences. Our analysis revealed the effect of newly occurring “non-hotspot” gene variants accompanying tumorigenesis progression in the example of hematological malignancies. Our study further expands our overall understanding of *DICER1* potential in neoplastic development. In the future, it could be valuable to expand such analysis to other oncology-associated genes and their inconclusive variants to develop the flexible methodology of variant evaluation in order to examine their potential effect with an appropriate set of instruments that could be adjusted individually for each marker.

## Data Availability

The original contributions presented in the study are included in the article/Supplementary Material; further inquiries can be directed to the corresponding author.
