# Corrigendum: Shedding light on the *DICER1* mutational spectrum of uncertain significance in malignant neoplasms

**DOI:** 10.3389/fmolb.2024.1517143

**Published:** 2024-11-28

**Authors:** D. S. Bug, I. S. Moiseev, Yu. B. Porozov, N. V. Petukhova

**Affiliations:** ^1^ Bioinformatics Research Center, Pavlov First Saint Petersburg Medical State University, St. Petersburg, Russia; ^2^ R. M. Gorbacheva Scientific Research Institute of Pediatric Hematology and Transplantation, Pavlov First Saint Petersburg State Medical University, St. Petersburg, Russia; ^3^ St. Petersburg School of Physics, Mathematics, and Computer Science, HSE University, Saint Petersburg, Russia; ^4^ Advitam Laboratory, Belgrade, Serbia

**Keywords:** Dicer1, variant of uncertain significance, variant effect prediction, gene evolution, oncology, molecular dynamics

In the published article, there was an error in Figure 7 as published. The Figures 7, 8 were mixed up. The corrected Figure 7 and its caption appear below.

**FIGURE 7:**
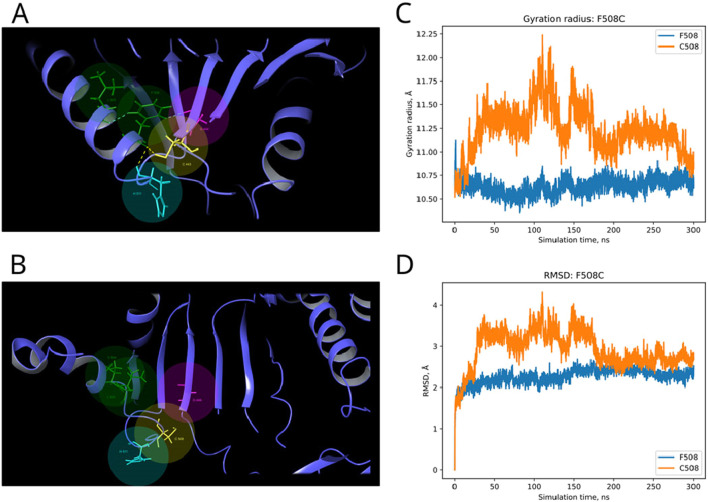
Structural alterations of Dicer1 variant F508C. **(A)** Interactions formed by wild-type amino acid F508. **(B)** Interactions formed by mutation C508. Amino acids taking part in bond formation are marked by spheres. H-bonds are indicated by dashed yellow lines, and aromatic H-bonds are indicated by dashed blue lines. Protein secondary structural elements (α-helixes, β-strands, and disordered loops) are shown in blue by cartoon representation. The radius of gyration **(C)** and RMSD **(D)** fluctuations of the 10 Å region around the wild-type amino acid and corresponding mutation through a 300-ns MD simulation.

In the published article, there was an error in Figure 8 as published. The Figures 7, 8 were mixed up. The corrected Figure 8 and its caption appear below.

**FIGURE 8:**
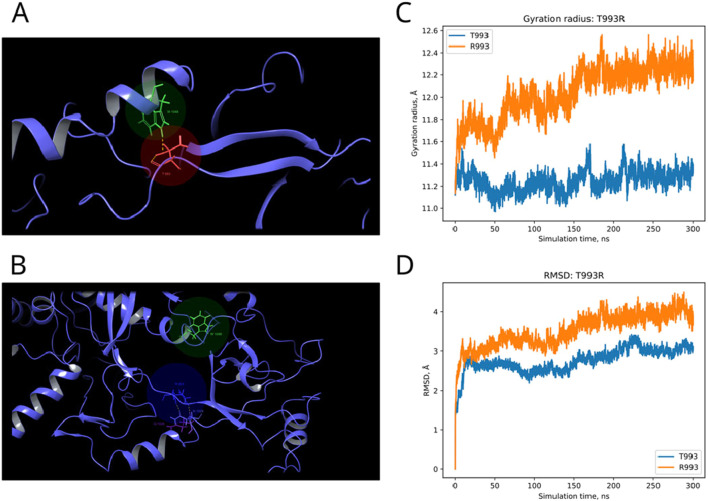
Structural alterations of Dicer1 variant T993R. **(A)** Interactions formed by wild-type amino acid T993. **(B)** Interactions formed by mutation R993. Amino acids taking part in bond formation are marked by spheres. H-bonds are indicated by dashed yellow lines, and aromatic H-bonds are indicated by dashed blue lines. Protein secondary structural elements (α-helixes, β-strands, and disordered loops) are shown in blue by cartoon representation. The radius of gyration **(C)** and RMSD **(D)** fluctuations of the 10 Å region around the wild-type amino acid and corresponding mutation through a 300-ns MD simulation.

The authors apologize for these error and state that this does not change the scientific conclusions of the article in any way. The original article has been updated.

