# Supplementary material for: Shedding light on the DICER1 mutational spectrum of uncertain significance in malignant neoplasms
Source: Front Mol Biosci. 2024 Oct 3;11:1441180. doi: 10.3389/fmolb.2024.1441180 (PMC11484276; doi:10.3389/fmolb.2024.1441180)
Supplement: Supplementary file 1 [file DataSheet2.PDF]

Supplementary Material

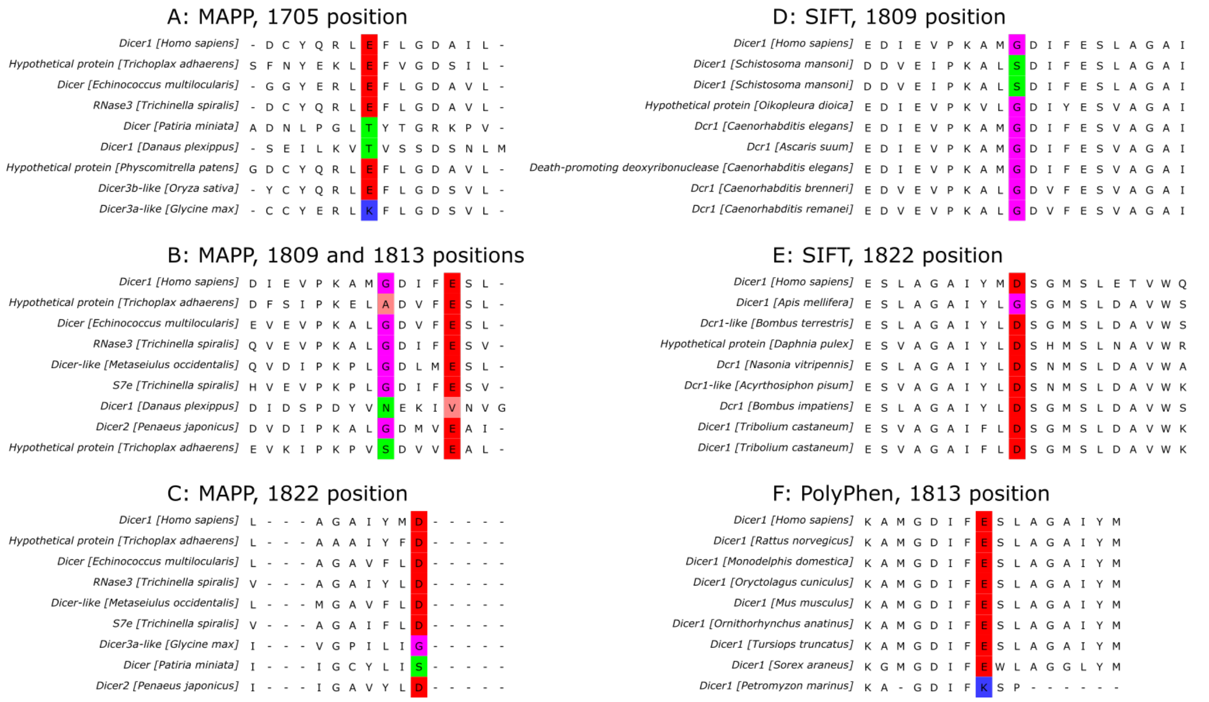

Supplementary Figure 1. Multiple sequence alignments used for variant effect prediction by MAPP (A, B, C), SIFT (D, E) and PolyPhen (F). Positions of known pathogenic mutations are highlighted.

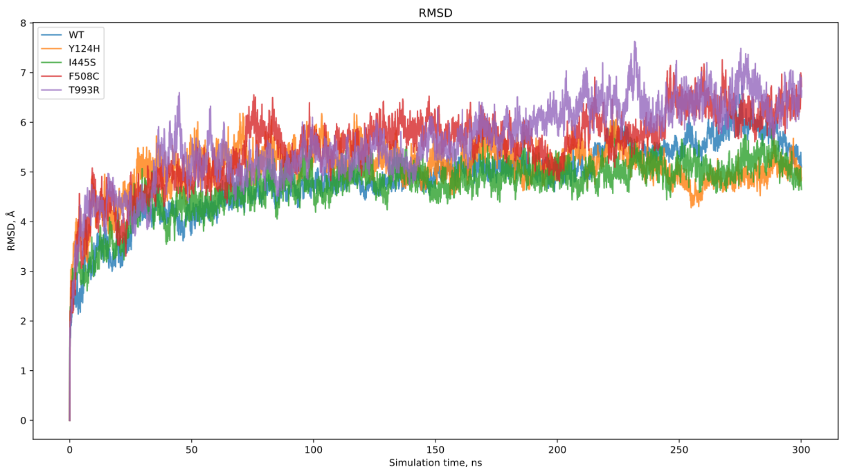

Supplementary Figure 2. Results of 300 ns MD simulations of Dicer wild-type protein along with its mutants (Y124H, I445S, F508C, T993R). RMSD trajectories of a protein backbone relative to the initial frame.

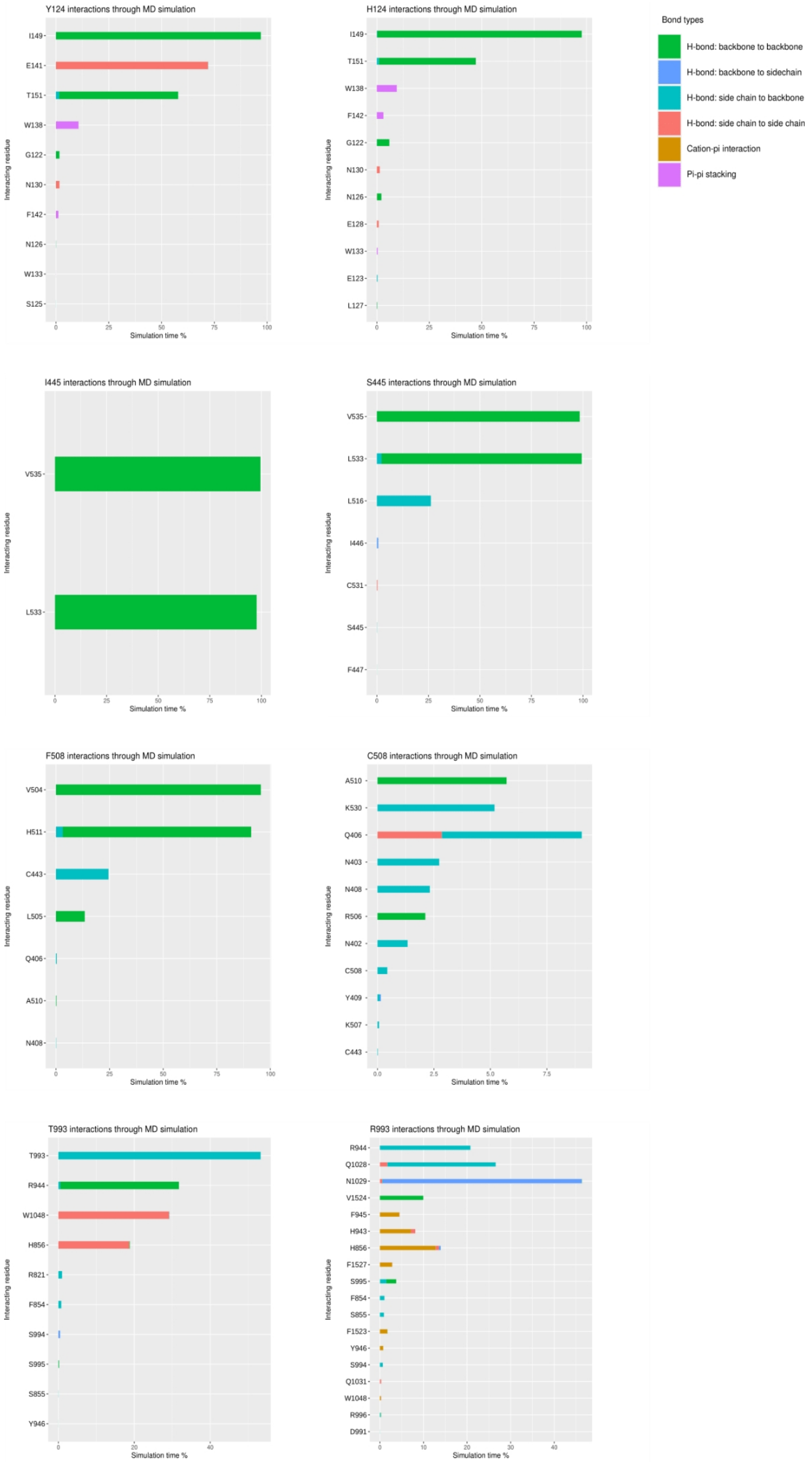

Supplementary Figure 3. Interaction analysis of WT and mutated residue through the 300 ns MD run.

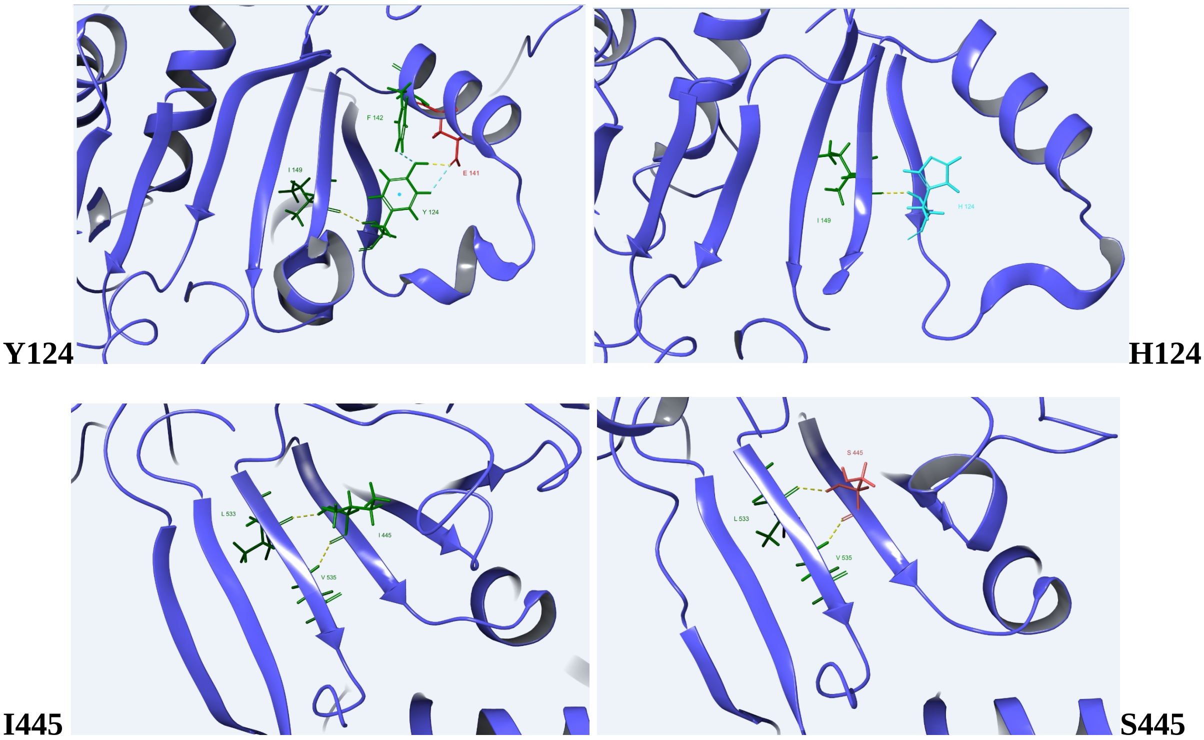

Supplementary Figure 4. Structural alterations of Dicer1 variant Y124H and I445S. H-bonds are indicated by yellow dash lines.

Supplementary Table 1. *In silico* prediction and evolutionary permissibility of studied variants according to different multiple sequence alignments of Dicer1 homologs.

**Variant**	**Observation**	**Evolutionary permissibility**	**Tools voting for variant benignity**	**Tools voting for variant pathogenicity**
Y124H	His is not present in any Metazoan species. In a single Vertebrate species Tyr is replaced with Cys	Not permitted	PolyPhen-1, SIFT, PhD-SNP	PolyPhen-2, MAPP, PANTHER, PredictSNP, SNAP
I445S	Only aliphatic amino acids are allowed (Ile, Leu and Val) in Metazoan species, Ile and Val are present in Vertebrate species	Not permitted	SNAP	PolyPhen-1, PolyPhen-2, SIFT, MAPP, PANTHER, PredictSNP, PhD-SNP, EVE
F508C	Cys is not present in any Metazoan species, Phe is conserved in Vertebrate species	Not permitted	-	PolyPhen-1, PolyPhen-2, SIFT, MAPP, PANTHER, PredictSNP, PhD-SNP, EVE, SNAP
T993R	Arg is not present in any Metazoan species, Thr is conserved in Vertebrate species	Not permitted	SIFT, PhD-SNP	PolyPhen-1, PolyPhen-2, MAPP, PANTHER, PredictSNP, EVE, SNAP

Supplementary Table 2. Statistics of MD simulations. Intra-protein H-bonds (HBonds_Intra), radius of gyration (rGyr), RMSD values of protein backbone relative to the initial frame, mean total energy of the system are presented.

	HBonds_Intra (count)		rGyr (Å)		RMSD_Backbone (Å)		Mean total E (kcal/mol)
	Range	Median	Range	Mean	Range	Mean	
**WT**	[1595, 1778]	1683	[44.713, 64.470]	47.454	[0.000, 6.761]	4.886	-55455.54
**Y124H**	[1607, 1782]	1687	[44.734, 64.537]	47.941	[0.000, 6.253]	5.143	-55451.89
**I445S**	[1619, 1775]	1690	[45.139, 60.238]	49.198	[0.000, 5.969]	4.744	-55563.06
**F508C**	[1579, 1779]	1657	[45.806, 64.844]	49.563	[0.000, 7.288]	5.553	-55338.9
**T993R**	[1589, 1794]	1656	[45.860, 57.025]	49.425	[0.000, 7.648]	5.638	-55188.02

Supplementary Table 3. MD simulation statistics of the 10 Å region around the residue in WT and mutated proteins. RMSD values of protein backbone, H-bonds between selected amino acids (H-bonds), radius of gyration (rGyr), and the surface area of selected regions are presented.

	**RMSD (Å)**	**Mean**	**H-bonds**	**Median**	**rGyr (Å)**	**Mean**	**Area (Å^2)**	**Amino acids selection**
**Y124**	[0.000, 2.701]	1.930	[42, 64]	54	[10.94, 11.54]	11.191	1864.01	res.num 89, 95, 96, 97, 98, 99, 100, 101, 102, 103, 104, 105, 106, 107, 108, 109, 110, 111, 113, 114, 120, 121, 122, 123, 124, 125, 126, 127, 128, 129, 130, 133, 134, 135, 136, 137, 138, 139, 140, 141, 142, 143, 144, 145, 146, 147, 148, 149, 150, 151, 152, 153, 154, 155, 156, 157, 158, 159, 163, 164, 169, 172
**H124**	[0.000, 2.838]	1.945	[41, 66]	53	[10.88, 11.54]	11.158	1906.01	
delta: mut-WT		*0.015*		*-1*		*-0.033*	*42*	
**I445**	[0.000, 2.157]	1.808	[43, 67]	55	[11.04, 11.33]	11.179	1070.57	res.num 37, 243, 250, 380, 383, 384, 387, 406, 441, 442, 443, 444, 445, 446, 447, 448, 449, 454, 457, 461, 474, 475, 476, 477, 478, 479, 480, 504, 505, 508, 509, 511, 513, 514, 515, 516, 517, 518, 519, 520, 521, 522, 523, 524, 526, 527, 528, 529, 530, 531, 532, 533, 534, 535, 536, 537, 538, 547, 548, 549, 550, 551, 552, 553, 554, 555, 558, 559, 560, 561, 562, 563
**S445**	[0.000, 4.005]	2.375	[46, 66]	56	[11.00, 11.34]	11.16	918.29	
delta: mut-WT		*0.567*		*1*		*-0.019*	*-152.28*	
**F508**	[0.000, 2.692]	2.264	[38, 62]	49	[10.35, 11.12]	10.634	1394.35	res.num 384, 399, 402, 403, 406, 407, 408, 409, 410, 441, 442, 443, 444, 445, 446, 447, 474, 475, 476, 477, 478, 479, 480, 500, 501, 502, 503, 504, 505, 506, 507, 508, 509, 510, 511, 512, 513, 514, 515, 516, 517, 518, 522, 526, 527, 528, 529, 530, 531, 532, 533, 534, 535, 552, 553, 554, 555, 558
**C508**	[0.000, 4.322]	2.921	[32, 57]	44	[10.52, 12.24]	11.235	1253.00	
delta: mut-WT		*0.657*		*-5*		*0.601*	*-141.35*	
**T993**	[0.000, 3.422]	2.756	[27.000, 48.000]	38	[10.97, 11.58]	11.244	1184.51	res.num 775, 790, 791, 792, 793, 794, 820, 821, 822, 853, 854, 855, 856, 857, 858, 929, 930, 931, 932, 933, 934, 942, 943, 944, 945, 946, 947, 948, 989, 990, 991, 992, 993, 994, 995, 996, 997, 998, 999, 1031, 1033, 1040, 1041, 1042, 1043, 1044, 1045, 1046, 1047, 1048, 1049, 1050, 1051, 1052, 1053, 1054, 1055, 1374, 1521, 1522, 1523, 1524, 1526, 1527
**R993**	[0.000, 4.508]	3.523	[20, 45]	33	[11.14, 12.56]	12.041	1128.31	
delta: mut-WT		*0.767*		*-5*		*0.797*	*-56.2*	
