# Supplementary material for: Shedding light on the DICER1 mutational spectrum of uncertain significance in malignant neoplasms
Source: Front Mol Biosci. 2024 Oct 3;11:1441180. doi: 10.3389/fmolb.2024.1441180 (PMC11484276; doi:10.3389/fmolb.2024.1441180)
Supplement: Supplementary file 2 [file DataSheet1.PDF]

Supplementary file 1. Maximum-likelihood tree in Newick format

(NP_001258211.1:0.0000028404,(XP_004055696.1:0.0012687033,((XP_009247731.
1:0.0000028404,((XP_024087404.1:0.0000028404,(XP_024087402.1:0.0000023237
,NP_001382627.1:0.0396070509)97:0.0000026835)52:0.0132034145,XP_024087403
.1:0.0000026344)97:0.0113090897)97:0.0037923795,(((XP_003260983.1:0.00000
28404,(XP_030658493.1:0.0000028404,XP_030658492.1:0.0237767338)100:0.0000
022267)98:0.0000028404,(XP_031991027.1:0.0000028404,XP_031991032.1:0.0000
028404)100:0.0025476476)100:0.0032438890,((((((((((NP_001382629.1:0.0000
028404,(((XP_020037990.1:0.0000028404,(XP_020037997.1:0.0000024002,XP_020
037995.1:0.0127703956)100:0.0109323279)100:0.0299613738,(XP_042548400.1:0
.0026607936,(XP_012870879.1:0.0000028404,XP_012870891.1:0.0000028404)100:
0.0026674002)100:0.0870591037)100:0.0157120354,(((((NP_001231198.1:0.0100
305272,(XP_005068415.1:0.0000028404,XP_040598495.1:0.0013546735)76:0.0082
139434)76:0.0142063777,((((((XP_021081829.1:0.0247009565,(XP_028715226.1:
0.0034091883,(XP_042117060.1:0.0000028404,XP_042117059.1:0.0162108182)100
:0.0060472181)100:0.0257484310)67:0.0054491429,XP_038191919.1:0.009460687
7)59:0.0031920875,XP_041489087.1:0.0275052089)98:0.0405279782,(XP_0414890
85.1:0.0038633873,XP_005343570.1:0.0012875926)100:0.0051786013)59:0.00000
28973,XP_038191917.1:0.0077886619)73:0.0060448102,((XP_036062149.1:0.0000
028404,XP_036062152.1:0.0000028404)100:0.0108551548,(XP_028715221.1:0.003
3162953,(XP_006997089.1:0.0000028404,XP_042117058.1:0.0000028404)100:0.00
58400480)100:0.0098190199)100:0.0144039880)71:0.0001713610)96:0.003823197
7,(XP_021502688.1:0.0235603565,((((XP_021058231.1:0.0000028404,XP_0293970
36.1:0.0159826760)100:0.0099339823,((((NP_683750.2:0.0000028404,XP_036013
155.1:0.0000028404)79:0.0000028404,((XP_036013153.1:0.0000020850,XP_01717
0487.1:0.0000028404)63:0.0000023618,XP_017170488.1:0.0000028404)100:0.014
7314577)49:0.0000028404,XP_017170486.1:0.0112113589)100:0.0174601266,XP_0
21034238.1:0.0225424724)100:0.0161520419)100:0.0034825559,XP_031213508.1:
0.0250411232)100:0.0109137618,(((XP_034343924.1:0.0000028404,XP_034343925
.1:0.0080588049)100:0.0189902567,(XP_028620262.1:0.0000028404,XP_02862026
4.1:0.0000028404)100:0.0374788323)100:0.0137474289,(XP_032764219.1:0.0000
028404,((XP_008763126.1:0.0000028404,(XP_017449994.1:0.0000028404,XP_0389
69290.1:0.0000026764)100:0.0147066999)86:0.0000028404,XP_038969293.1:0.00
94130917)100:0.0078405239)100:0.0332236120)100:0.0026844600)100:0.0176295
027)100:0.0028718996)100:0.0417415348,(XP_008835795.1:0.0000028404,(XP_02
9421486.1:0.0000028404,(XP_029421485.1:0.0041760775,XP_017654713.1:0.0134
135391)66:0.0000023448)100:0.0083920508)100:0.0650968400)97:0.0125269938,
XP_004665669.1:0.1148324367)100:0.0213145850)96:0.0000027246)48:0.0000024
701,NP_001382628.1:0.0114185804)91:0.0147171454,((((XP_004837064.1:0.0000
023961,XP_021100324.1:0.0000028404)100:0.0168103252,(XP_010607463.1:0.000
0028404,XP_010607465.1:0.0318736632)100:0.0206087496)100:0.0091146246,(XP
_013372660.1:0.0156024802,(XP_004635228.2:0.0000021661,XP_023575370.1:0.0
000028404)100:0.0292207723)100:0.0057974798)100:0.0031869983,XP_003463041
.3:0.0286018752)100:0.0126027535)69:0.0041166317,((XP_027806737.1:0.00129
23076,XP_015344035.1:0.0079347250)100:0.0028114645,(XP_005341233.1:0.0000
028404,(XP_021576440.1:0.0000028404,((XP_026267567.1:0.0000028404,((XP_02
6267571.1:0.0055624451,XP_040131806.1:0.0025022481)100:0.0095373441,XP_02
6267566.1:0.0295933454)98:0.0197586490)94:0.0025872127,XP_040131807.1:0.0
012818940)39:0.0000023461)40:0.0000028743)99:0.0040221660)100:0.033739507
4)95:0.0102674924,(((XP_004584367.1:0.0000028404,XP_012782186.1:0.0956614
800)100:0.0070107143,XP_040831044.1:0.0051533021)100:0.0809958088,XP_0171
94768.1:0.1271658469)96:0.0438446519)78:0.0047890287,(((((((((XP_0089359
78.1:0.0000028404,((XP_034962559.1:0.0089176527,XP_032996278.1:0.00651742
57)97:0.0025494502,XP_028572674.1:0.0039868512)60:0.0022082041)40:0.04567
79495,((XP_007422466.2:0.0157364509,(((XP_039212144.1:0.0000028561,XP_039
212148.1:0.0000028404)100:0.0089914071,XP_015674552.1:0.0025530812)100:0.
0209565468,((XP_026535162.1:0.0013044983,XP_026572786.1:0.0037985620)100:
0.0153779627,(XP_034274297.1:0.0103134430,(XP_032089625.1:0.0012799594,XP
_013916564.1:0.0025670145)100:0.0141937643)100:0.0012815446)100:0.0062288
109)100:0.0314573580)98:0.0460469020,((XP_042320622.1:0.0399919174,XP_003

214365.1:0.0482747962)100:0.0201389583,XP_020666690.1:0.0930773249)98:0.0
156984275)84:0.0029799761)81:0.0145903192,XP_015271827.1:0.0435900201)91:
0.0852003228,((((XP_010016930.1:0.0000028404,XP_010204807.1:0.0000021685)
45:0.0507863350,((((((((((((((((XP_027494065.1:0.0000028404,((XP_027574
201.1:0.0000028404,(XP_017685320.1:0.0012728418,XP_029819959.1:0.00127307
54)99:0.0000028404)100:0.0012742431,XP_032548191.1:0.0038233078)99:0.0000
028404)99:0.0012741051,(XP_027748611.1:0.0000023961,((XP_027494068.1:0.00
11503567,(((XP_017685317.1:0.0000028404,(XP_017685324.1:0.0000028641,XP_0
17685323.1:0.0000024002)100:0.0126846305)100:0.0012122893,XP_027574205.1:
0.0012131273)100:0.0000028404,XP_029819962.1:0.0060950235)100:0.002513593
0)100:0.0181282607,XP_027748614.1:0.0025152639)100:0.0359701656)98:0.0040
559548)100:0.0054302231,(((((XP_032917720.1:0.0038274067,XP_005047604.1:0
.0050892134)53:0.0000028404,XP_014739947.1:0.0102285636)100:0.0025418906,
(((((XP_037995172.1:0.0051269275,XP_026646942.1:0.0025463075)58:0.0000028
404,((XP_036239497.1:0.0000028404,XP_036239499.1:0.0067520247)100:0.00130
64159,XP_030095680.1:0.0012729198)51:0.0000028561)31:0.0000028561,(XP_005
417921.1:0.0012743058,(NP_001156875.1:0.0064240218,XP_021405357.1:0.00896
45498)73:0.0012680329)72:0.0012726691)45:0.0000028561,(XP_039568420.1:0.0
038186271,(XP_041332158.1:0.0006881149,XP_041269954.1:0.0046297834)100:0.
0048968954)97:0.0012735015)100:0.0052283768,XP_039922070.1:0.0166145829)6
9:0.0000028561)69:0.0000028561,((XP_015485719.1:0.0025513089,(XP_02378338
7.1:0.0000028404,(XP_023783390.1:0.0000028404,XP_023782935.1:0.0000021362
)100:0.0516382861)100:0.0063734435)100:0.0012499882,XP_005520216.1:0.0038
232118)100:0.0089866792)100:0.0013120038,((XP_041902896.1:0.0012700632,XP
_031966629.1:0.0012688898)100:0.0000028404,(XP_010392939.1:0.0000028404,X
P_008627957.1:0.0012699095)100:0.0012691589)100:0.0064163524)100:0.006554
7785)100:0.0013101388,XP_009072525.1:0.0069300566)100:0.0066246368,XP_010
304722.1:0.0168540068)93:0.0026085355,(((((XP_009330655.1:0.0038068288,XP
_019328047.1:0.0323387558)100:0.0102510696,XP_014813068.1:0.0074884821)81
:0.0017156632,(((((XP_032043622.1:0.0025528738,XP_038036674.1:0.000002840
4)100:0.0025503914,((XP_035183124.1:0.0012737140,XP_013037157.1:0.0064002
122)61:0.0000028404,((XP_035395254.1:0.0000028404,XP_035395260.1:0.000002
8404)100:0.0025517583,XP_040414085.1:0.0012751013)100:0.0025491819)61:0.0
000028404)100:0.0097409669,((((((XP_031448903.1:0.0000028404,XP_031448906
.1:0.0263283084)99:0.0012720540,(((XP_042740729.1:0.0000028561,XP_0427411
39.1:0.0000028404)100:0.0038118937,(XP_042669423.1:0.0000598912,XP_042669
427.1:0.0028343614)100:0.0012103421)100:0.0025502922,XP_010710198.2:0.045
1089930)100:0.0025457753)62:0.0001208051,XP_010710189.1:0.0057187173)41:0
.0011519172,NP_001035555.1:0.0025438120)77:0.0000028561,XP_015720801.1:0.
0063858243)99:0.0044803648,XP_021259280.1:0.0084229959)100:0.0138310528)1
00:0.0110415329,((XP_009506932.1:0.0133566669,XP_010192237.1:0.0169843835
)95:0.0043884335,XP_009579824.1:0.0218300763)94:0.0000028404)94:0.0014320
813,XP_010161909.1:0.0185811683)79:0.0012059435)97:0.0013028147,(((XP_042
646730.1:0.0000028404,XP_042646736.1:0.0000028404)100:0.0000028404,XP_032
866398.1:0.0431791448)100:0.0116091555,XP_026705052.1:0.0210596998)99:0.0
025178590)90:0.0013013430,XP_009935118.1:0.0207139119)69:0.0000022288)50:
0.0000029331,XP_009869144.1:0.0246600624)50:0.0000028743,XP_010139397.1:0
.0271749991)90:0.0003249429,XP_009944268.1:0.0043553997)88:0.0009812629,X
P_010204994.1:0.0308145812)73:0.0000029323,((XP_005438920.1:0.0000028404,
XP_027651908.1:0.0105412336)100:0.0102960596,XP_009694185.1:0.0154462330)
78:0.0012527918)24:0.0000028743,(((XP_029895892.1:0.0000028404,XP_0409856
14.1:0.0039980928)65:0.0000028404,((XP_010582193.1:0.0000028404,XP_009910
593.1:0.0093757958)94:0.0001197901,XP_009926056.1:0.0000028404)95:0.00242
56994)92:0.0051621169,XP_008936018.1:0.0302886489)33:0.0000028404)30:0.00
00029331,(((XP_009807270.1:0.0180553506,(XP_009501036.1:0.0155813554,XP_0
09582534.1:0.0105666483)100:0.0376242791)98:0.0000020647,XP_009906182.1:0
.0162213885)87:0.0035210489,((XP_030337356.1:0.0055547748,XP_033917521.1:
0.0267736439)100:0.0025694489,XP_010020761.1:0.0132315798)100:0.015463019
4)80:0.0038226798)65:0.0013073294,XP_009557474.1:0.0181227592)26:0.000002
0409,((XP_010286683.1:0.0102849185,XP_009959863.1:0.0093916389)64:0.00000
23843,(XP_010190007.1:0.0110170567,XP_010148488.1:0.0155353867)80:0.00258

26534)36:0.0000028404)48:0.0000028743,(((XP_010710191.1:0.0000028404,(XP_
009977804.1:0.0167243995,(XP_010002039.1:0.0180496542,XP_030308110.1:0.02
46542520)100:0.0052723846)54:0.0000028786)25:0.0000028561,((XP_009638799.
1:0.0090482969,(XP_009484159.1:0.0102753786,XP_009465756.1:0.0115492975)9
4:0.0000028561)93:0.0012741805,XP_009879824.1:0.0128493390)41:0.000002840
4)46:0.0000598912,XP_009805123.1:0.0179884688)72:0.0012151148)54:0.001307
0103,(XP_010128284.1:0.0154389903,XP_005506174.1:0.0180750933)86:0.000002
8404)50:0.0013084007,XP_010085136.1:0.0221235987)46:0.0053851781)76:0.008
7924570,(((XP_009575579.1:0.0000028404,((XP_025963471.1:0.0000028404,XP_0
25963473.1:0.0053356412)100:0.0039405091,((XP_025902760.1:0.0000028404,XP
_025902768.1:0.0013157998)100:0.0140949924,XP_010217920.1:0.0155880106)92
:0.0064461796)44:0.0000598912)68:0.0011140677,((XP_013796167.1:0.00000284
04,XP_025935245.1:0.0000028404)100:0.0000028404,(XP_025935256.1:0.0004730
362,XP_009081478.1:0.0000028404)100:0.0113747552)100:0.0076972459)80:0.00
12841623,(XP_009688039.1:0.0000028404,XP_009688036.1:0.0118745236)100:0.0
206073434)88:0.0054846821)93:0.0309614519,((XP_019374155.1:0.0064056055,X
P_019408008.1:0.0051201336)100:0.0089091579,(XP_006275891.1:0.0025940121,
XP_025062058.1:0.0089977781)100:0.0054321956)100:0.0406247489)95:0.010058
0019)89:0.0057985761,((((XP_039390679.1:0.0048764928,(XP_032627944.1:0.01
17263648,XP_030414687.1:0.0152233333)100:0.0086676464)100:0.0019996605,(X
P_024075105.1:0.0037888357,(XP_034623922.1:0.0025271725,((XP_005307563.1:
0.0000028404,XP_042714609.1:0.0000028404)100:0.0001107003,XP_042714610.1:
0.0000028404)100:0.0036925201)100:0.0050692060)100:0.0045539803)100:0.002
8821867,((XP_037756814.1:0.0000021147,XP_043404338.1:0.0110094978)99:0.01
00823591,(XP_038261433.1:0.0000024002,XP_043371923.1:0.0056785794)99:0.01
25460084)100:0.0047007362)100:0.0088536301,XP_006133754.1:0.0330797604)10
0:0.0338307155)97:0.0102589480,((((XP_006003961.1:0.0000028404,XP_0143486
97.1:0.0000028561)100:0.2149267919,((((((XP_041070186.1:0.0191027573,XP_
038678239.1:0.0483527337)96:0.0052303805,(((XP_043552850.1:0.0000028404,X
P_043552854.1:0.0095921110)100:0.0000028685,(XP_043552855.1:0.0000027450,
XP_043552856.1:0.0168822085)100:0.0326985233)100:0.0229431214,XP_02038579
0.1:0.0203450267)100:0.0307255908)96:0.0433673815,XP_032883417.1:0.121006
5436)96:0.0377932880,(XP_020516781.1:0.0000028404,XP_010790812.1:0.093888
8090)99:0.1707815653)98:0.0335589204,(XP_007909546.1:0.0012799731,XP_0421
93799.1:0.0000028685)100:0.1135473342)99:0.2595457325,(XP_032820916.1:1.1
792955256,(((XP_035677529.1:0.0846882805,(XP_019642793.1:0.0000028404,XP_
019642797.1:0.0000028404)100:0.1199381912)100:1.5285106167,((XP_006824151
.1:1.4155331006,((XP_033114296.1:0.0000028404,XP_033114295.1:0.0000026371
)100:1.4942657538,(((XP_022096692.1:0.3735786065,XP_038077645.1:0.2936195
347)100:0.3212707305,XP_033634026.1:0.5887915751)100:0.7833703375,((XP_04
1468155.1:0.0000028404,XP_041468153.1:0.0000028404)100:0.2382097439,XP_01
1668948.2:0.1980625205)100:1.2601916050)100:0.6143094653)100:0.5090127020
)100:0.3658227642,((XP_013409876.1:1.3195563533,(((((XP_022331683.1:0.235
4280249,XP_011439629.2:0.2180408829)100:0.8949040922,(XP_021347347.1:0.23
91343263,XP_033733992.1:0.2586690972)100:1.0398149289)100:0.4092996972,((
XP_009046342.1:1.0118819503,XP_041353083.1:0.9545931145)96:0.1075545548,(
XP_025104500.1:1.0350179894,(XP_012942349.1:0.5984730423,XP_013067888.1:0
.6241636718)100:0.8229570732)100:0.2587431702)100:0.2324848999)98:0.16091
85927,((XP_029642569.1:0.0067164303,XP_029648589.1:0.0149832201)100:0.014
9763387,XP_014781473.1:0.0143061428)100:1.4350307283)100:0.4445276879,XP_
009029520.1:2.3326204064)99:0.1698731766)100:0.6965937482,(((((((XP_04289
5187.1:0.0000028641,XP_042895189.1:0.0000028404)100:0.6008711201,XP_03523
1209.1:0.5882657285)100:0.8031116338,(((XP_022250670.1:0.4994729732,(XP_0
22241304.1:0.0000028404,XP_013774216.2:0.0000020529)100:1.3749281698)100:
0.5692851083,XP_023219964.1:1.0113922164)100:0.1768018743,((XP_042144649.
1:0.3823859667,(XP_037575705.1:0.0949542110,(XP_037499367.1:0.0387753344,
XP_037275673.1:0.1174951857)100:0.0781863130)100:0.3033513053)100:0.90163
34730,((((XP_022665652.1:0.0198061993,XP_022665643.1:0.0000028404)100:0.0
023434544,XP_022704558.1:0.0000022911)100:0.4566780540,XP_018495704.1:0.7
050810722)100:1.0258998994,(XP_027197063.1:1.1178653311,XP_017461388.1:0.
4472091849)100:2.1913813906)100:0.8745680322)100:0.6668313223)93:0.138424

0724)100:0.5642799163,XP_015789823.1:3.4223651421)99:0.2707763997,((((XP_
042234733.1:0.2912234854,((XP_027219408.1:0.0226297959,XP_037797435.1:0.0
203399537)100:0.0362137588,XP_042886929.1:0.0307367909)100:0.2833573519)1
00:0.6641026775,XP_018026822.1:1.3282717718)100:0.9328301791,(XP_03708770
1.1:0.5301187881,XP_043238115.1:0.5731698720)100:1.5283837754)100:0.33306
62667,(((((((((((XP_020299785.1:0.2605295283,XP_012216792.1:0.1163388864
)96:0.0197465207,(((XP_011257654.1:0.0643518584,XP_029662169.1:0.04566351
23)100:0.0290122129,XP_029166413.1:0.0594596744)100:0.0657600807,(((XP_01
2529231.1:0.0729547550,XP_025991666.2:0.0749538530)100:0.0402946111,(((XP
_011881534.1:0.0808368622,XP_024871879.1:0.0850348450)100:0.0099750118,XP
_024870808.1:0.1205106913)100:0.0268935834,(((XP_018318341.1:0.0293764230
,(((XP_018346016.1:0.0259527038,(XP_012055107.1:0.0039870025,XP_018053528
.1:0.0031587377)100:0.0207414611)100:0.0052281923,XP_011062325.1:0.017819
9713)100:0.0099077366,XP_018360760.1:0.0134650020)100:0.0056072542)100:0.
0177794659,XP_018396309.1:0.0620456866)100:0.0215802876,(XP_011689343.1:0
.0000028561,XP_011689345.1:0.0054455952)100:0.0616949523)93:0.0123750749)
93:0.0045880388)100:0.0180201804,XP_025074738.1:0.1245494744)100:0.051185
3588)100:0.0197639596)100:0.0228034928,XP_011344594.1:0.1820733671)100:0.
0809326440,(XP_032687320.1:0.1006659205,(XP_011149855.1:0.1061899971,XP_0
14467345.1:0.1111395343)94:0.0201611761)100:0.0916929977)100:0.1575943244
,((((((((((NP_001116485.2:0.0029003869,XP_016912117.1:0.0131318611)87:0.
0052117029,(XP_016905551.1:0.0000028404,XP_028520499.1:0.0337223342)99:0.
0115210134)55:0.0004793435,XP_016912146.1:0.0000028404)58:0.0035741134,(X
P_006618601.1:0.0084733962,XP_043793231.1:0.0046612562)79:0.0035044564)10
0:0.0077938799,XP_003696618.3:0.0253833587)100:0.1091979961,XP_017754466.
1:0.1468726850)100:0.0136911998,((((XP_043603978.1:0.0000028404,XP_043603
979.1:0.0011949385)100:0.0233732373,(((XP_033360236.1:0.0000028404,XP_033
360235.1:0.0000028404)100:0.0072950642,(XP_033179510.1:0.0000028404,XP_03
3179509.1:0.0000028404)100:0.0060929679)66:0.0000028404,(XP_033205433.1:0
.0000028404,XP_033205432.1:0.0000028404)100:0.0048648717)100:0.0200350407
)99:0.0090669641,XP_020723074.1:0.0278112687)100:0.0753534548,(XP_0435228
30.1:0.0000028404,XP_043522831.1:0.0085958874)100:0.1124525370)100:0.0317
111486)100:0.0739726913,XP_017788557.1:0.1774929917)100:0.0348153179,((XP
_012148052.1:0.0069529363,XP_003706496.1:0.0000021685)100:0.1200156002,(X
P_034184897.1:0.0159782949,XP_029033305.1:0.0000023961)100:0.0576143530)1
00:0.1487280230)100:0.0324260567,(((XP_033332973.1:0.2538179170,XP_031838
404.1:0.1097915948)100:0.0711437799,XP_015439626.1:0.1729042753)100:0.100
5437726,XP_043263545.1:0.1963175643)100:0.0624204943)100:0.0734100183,XP_
017892073.1:0.9621232172)95:0.1129160436)57:0.0413936088,((XP_043680723.1
:0.0916315533,XP_035741937.1:0.0603645146)100:0.1431669040,(XP_015185292.
1:0.3087732433,(((XP_043489189.1:0.0000028404,XP_043489190.1:0.0000028404
)80:0.0000028404,XP_043489191.1:0.0000028404)100:0.1042409687,((XP_014605
687.1:0.0000028404,((XP_014605688.1:0.0000028404,XP_014605691.1:0.0000028
404)84:0.0000028404,XP_014605689.1:0.0000028404)33:0.0000028404)72:0.0000
024002,XP_014605690.1:0.0000028404)100:0.1337751255)99:0.0975394079)100:1
.0560915348)100:0.1939262970)100:0.1239166840,((((XP_015607218.1:0.000002
8404,XP_015607216.1:0.0000028404)100:0.2653427433,(XP_015513890.1:0.28244
29188,XP_012254499.1:0.2305203856)100:0.4956792839)99:0.0924389914,XP_012
286436.1:0.4804112102)64:0.0669854983,(XP_033227813.1:0.2206293767,XP_043
480045.1:0.3107305733)100:0.3531801470)57:0.0493694609)94:0.1400315303,((
((XP_014296866.1:0.0000022776,XP_014296868.1:0.0012797673)100:0.410050052
0,XP_034941920.1:0.3220940774)100:0.1945734431,((XP_015125778.1:0.1696827
978,XP_011315268.1:0.2147438303)100:0.3875219868,XP_044014882.1:0.7816443
099)100:0.1251453326)100:0.3051272078,(XP_043278021.1:0.0000028685,XP_043
278022.1:0.0000028404)100:0.6485674730)100:0.1035539806)100:0.3051311303,
(((XP_001605287.1:0.3666841710,(XP_011502152.1:0.0000023824,XP_011502153.
1:0.0034624136)100:0.4450975347)100:0.2920842520,XP_014210593.1:0.8376729
666)100:0.1627119471,XP_014235184.1:1.4861543090)100:0.2263903025)100:0.6
942336323,(((((XP_026287017.1:0.2974917914,XP_034252742.1:0.3331019760)10
0:0.9890266534,(((((XP_021929030.1:0.0031440367,XP_021929006.1:0.00000284
04)100:0.0037506532,XP_021929008.1:0.0032389746)61:0.0000418633,XP_021929

010.1:0.0000028404)97:0.0008886459,XP_021929009.1:0.0000028501)100:0.1994
746812,XP_023719035.1:0.2291927475)100:0.5968678192)100:0.2445560492,XP_0
02429494.1:1.5017975617)98:0.1317862380,((XP_039288314.1:0.9885057668,(XP
_014250997.1:0.7111825458,XP_014270680.1:0.6185938723)100:0.6752296433)10
0:0.2075161811,((((((XP_026808418.1:0.0757980831,XP_027837610.1:0.0639788
117)100:0.0220619953,XP_025195099.1:0.1119651732)100:0.0451948355,((XP_01
5363113.1:0.0000028641,(XP_015363115.1:0.0000028404,XP_015363114.1:0.0000
598912)100:0.0013539891)100:0.0875512835,((XP_022183763.1:0.0515132876,(X
P_029346748.1:0.0114113105,XP_029346747.1:0.0000028404)100:0.0689804044)7
5:0.0072289983,XP_029341322.1:0.4683493298)75:0.0069187460)100:0.05396748
73)100:0.1722371613,(XP_025420847.1:0.0000020514,XP_025420848.1:0.0000028
404)100:0.2303474099)100:1.3937405381,XP_026677341.1:1.9497335655)100:0.2
209255530,XP_018901898.1:1.2600970415)100:0.2341527476)100:0.1892411905)1
00:0.1363288824,((((XP_031344814.1:0.8695534663,(XP_018328602.1:0.0000024
663,XP_018328603.1:0.0000028404)100:0.8069506404)100:0.1960627602,(((((XP
_023016629.1:0.5766837336,((XP_028151555.1:0.0000025594,XP_028151556.1:0.
0000028404)98:0.1435017201,XP_028151557.1:0.0931770056)100:0.3914913096)1
00:0.1059517553,XP_018576096.1:0.3473552990)100:0.1374369461,(XP_03076229
8.1:0.4197648166,XP_019765036.1:0.5210635956)100:0.3918829077)100:0.11878
81723,XP_019877739.1:0.7126423225)100:0.1570442716,XP_008199045.1:0.58437
76341)100:0.3279279239)100:0.0906627342,(XP_022902072.1:0.8042581395,XP_0
17783677.1:1.3220942025)99:0.2079513080)100:0.8348653167,((((XP_037911100
.1:0.7573726906,((((((((XP_011212659.1:0.0147267018,XP_018784623.1:0.0324
969543)100:0.0012443163,XP_039970434.1:0.0126513068)100:0.0429404947,XP_0
14088091.1:0.0603342106)100:0.0647835907,XP_011185944.1:0.1180317027)100:
0.1426830045,(XP_004523313.1:0.1972018470,(XP_036323127.1:0.0116771861,XP
_017475087.1:0.0063320119)100:0.2323923230)90:0.0518737727)100:0.29998492
09,((XP_037945044.1:0.0000023961,XP_037943688.1:0.0082806255)100:0.004065
0431,XP_037943698.1:0.0050372986)100:0.5327785227)100:0.1337385188,(XP_03
7891626.1:0.4154275026,((XP_013119344.1:0.2748169065,XP_005179923.1:0.291
9325362)100:0.1250726200,(XP_037818632.1:0.0333854233,XP_023292790.1:0.03
87520972)100:0.2967746926)100:0.1441803664)100:0.3610343589)100:0.1242803
380,(((((((((XP_017874916.1:0.0000028404,XP_017874914.1:0.0000022856)100
:0.0138713988,XP_001999145.1:0.0158673221)100:0.0094697016,XP_030239877.1
:0.0454647829)100:0.0664794056,XP_030080628.1:0.0773533057)100:0.10696170
62,(XP_030573569.1:0.0343766422,XP_002054279.1:0.0315770331)100:0.0840605
905)100:0.0779362246,XP_001989969.1:0.1809871398)100:0.0618387500,XP_0344
85812.1:0.1409681101)100:0.0507591430,XP_017848749.1:0.4028292369)100:0.1
256559172,((((((((XP_017082697.2:0.1200498179,((XP_037712636.1:0.0161919
080,XP_036672987.1:0.0251185504)100:0.0307859258,XP_016960843.1:0.0546324
426)100:0.0331640782)64:0.0084892029,((((XP_002098342.1:0.0118515266,XP_0
39493259.1:0.0082582137)100:0.0083239505,XP_043656235.1:0.0082453065)100:
0.0148491813,XP_001982226.1:0.0259368984)100:0.0140764948,((XP_002104476.
2:0.0173274151,(XP_032578931.1:0.0172075894,XP_033164246.1:0.0069088136)1
00:0.0015649412)100:0.0076589908,NP_524453.1:0.0262567910)100:0.031348765
0)100:0.0737785263)64:0.0098895470,XP_017003684.1:0.0835762100)100:0.0112
089464,(XP_016974488.1:0.0583391505,XP_017114515.1:0.0583778480)100:0.038
7336953)100:0.0211922557,XP_017052543.1:0.1153933881)100:0.0469373448,(XP
_017035067.1:0.0716728055,XP_020799646.1:0.0625931504)100:0.1159467788)10
0:0.0506768617,(XP_001954492.2:0.0768534966,XP_017101968.2:0.0650939047)1
00:0.1707576483)100:0.0506849633,(((((XP_034131095.1:0.0021029456,((((XP_
034131087.1:0.0000028404,XP_034131088.1:0.0000028404)98:0.0000028404,(XP_
034131085.1:0.0000028404,(XP_034131086.1:0.0000028404,(XP_034131089.1:0.0
000028404,(XP_034131090.1:0.0000028404,XP_034131091.1:0.0000028404)95:0.0
000028404)95:0.0000028404)95:0.0000028404)82:0.0000028404)94:0.0000022809
,XP_034131094.1:0.0058642800)37:0.0030651117,(((XP_034662290.1:0.00000284
04,(XP_034662293.1:0.0000028404,XP_034662292.1:0.0000028404)94:0.00000284
04)36:0.0000028404,(XP_034662291.1:0.0000028404,(XP_034662294.1:0.0000028
404,(XP_034662295.1:0.0000028404,XP_034662296.1:0.0000028404)100:0.000002
8404)100:0.0000028404)92:0.0000028404)98:0.0000023961,XP_034662297.1:0.00
00028404)100:0.0445979904)47:0.0172645967)100:0.4411294658,XP_034131092.1

:0.0000028561)100:0.0241527065,XP_034131093.1:0.0000028561)100:0.02201940
31,XP_034662298.1:0.0172586144)100:0.0378299245,(((XP_002012751.1:0.00394
20241,XP_001358121.2:0.0072109285)100:0.0010870648,XP_017142899.1:0.02340
34016)100:0.0866468212,XP_041452392.1:0.0669646268)92:0.0188491016)100:0.
1874240112)100:0.0849072507,XP_002069632.1:0.3691853937)100:0.0685087797)
100:0.1467492160,XP_030371583.1:0.3026326073)100:0.5091914361)100:0.58925
21703)100:0.1695276735,((XP_037027716.1:0.0000028404,XP_037027715.1:0.000
0028641)100:0.7798748531,XP_031628056.1:1.0157783429)100:0.1794521736)100
:0.1357502901,(((((XP_029717928.1:0.0000028404,XP_029717929.1:0.000002840
4)100:0.0121387874,XP_029717846.1:0.0137287078)100:0.1030226478,XP_001659
747.2:0.1287559316)100:0.1871795680,XP_039438687.1:0.3051716349)100:0.432
3971755,((XP_035894675.1:0.2394084987,((XP_041785729.1:0.0160390163,((XP_
003436256.1:0.0000028404,XP_312076.2:0.0000028404)100:0.0054707685,XP_040
227609.1:0.0050543989)100:0.0021241689)100:0.0029054495,XP_040160693.1:0.
0090031406)100:0.2684847347)100:0.2261300568,XP_035780167.1:0.3940642552)
100:1.1049811734)100:0.2720634610)100:0.4239609826,(((((((((((XP_0264864
19.1:0.3004935512,XP_032528041.1:0.4328893207)100:0.0809647704,(XP_034838
755.1:0.3079640027,(XP_023938455.1:0.3152678324,(XP_039762549.1:0.0000028
404,XP_039762547.1:0.0000028404)100:0.3760941463)100:0.0826660475)100:0.1
811538472)100:0.0832674945,XP_041984261.1:0.5816603804)100:0.0492122254,X
P_037972607.1:0.3256903466)63:0.0185675100,(XP_022123463.1:0.4209021498,X
P_038220360.1:0.4913040696)97:0.2149491907)71:0.1530367417,(((XP_02632819
6.1:0.2720577994,XP_028160127.1:0.3762291296)84:0.0449600252,XP_013188945
.1:0.6287672252)78:0.0698675346,(((XP_014366247.1:0.0606933738,XP_0131634
21.1:0.1283548056)100:0.0249831962,XP_013141891.1:0.1293400327)99:0.07984
09971,XP_014370155.1:0.4329880414)100:0.3221251948)70:0.1021198578)95:0.0
530679640,((XP_026747931.1:0.3372599739,((XP_022832341.1:0.0888397003,XP_
035457172.1:0.0734988564)100:0.1609511540,XP_021195706.1:0.1829828520)100
:0.1582084135)97:0.2078123086,((XP_026749666.2:0.5218456955,((XP_03729664
1.1:0.0000021331,(XP_037302645.1:0.0058593373,XP_037301670.1:0.0246936427
)100:0.0140995865)100:0.1032892944,XP_037302492.1:0.1465573681)100:0.2793
241708)86:0.1270874404,(XP_028040138.1:0.0181533138,((XP_037869731.1:0.00
00028404,XP_037869733.1:0.0000028404)51:0.0000028404,XP_037869732.1:0.000
0028404)100:0.0062911147)100:0.6772815346)87:0.1276331072)90:0.0635696044
)99:0.2260883646,(XP_021199950.1:0.4850115864,XP_031767635.1:0.6492013297
)99:0.0894251590)85:0.0304004566,XP_028160183.1:0.6368566115)86:0.0718028
810,XP_037972432.1:0.7157271513)95:0.1447244945,XP_032528124.1:0.67937406
32)100:1.4395054246,XP_026470854.1:1.6212810371)96:0.2451366443)96:0.1708
907376)94:0.0909699542)94:0.1449485285)100:0.2121295355,(((XP_040583376.1
:0.0077176323,XP_040582954.1:0.0027437672)100:1.5627690187,XP_023324353.1
:1.6777529562)100:0.6894016543,XP_021966369.1:2.3601093201)99:0.312497218
5)99:0.3994962688,XP_032798933.1:1.9692399634)97:0.2067851415)100:0.41468
03547)100:0.2810143499,(((((XP_040076155.1:0.0523923143,XP_040076154.2:0.
0201100460)100:0.7966730463,(((XP_037562843.1:0.1408814167,(XP_037576626.
1:0.0136499528,(XP_037576623.1:0.0000022936,XP_037557660.1:0.0054695699)1
00:0.2318656912)100:0.0530592103)100:0.2292214015,(XP_037274583.1:0.21048
06829,XP_037502218.1:0.1440641555)99:0.1398397092)81:0.0708672606,XP_0375
01243.1:0.0566428303)100:0.7405925153)100:2.6016395619,((XP_015914875.2:1
.3816166647,(XP_035226094.1:0.5717877235,(XP_035222013.1:0.0000028404,XP_
035222012.1:0.0000028714)100:1.1293715965)100:0.7351683414)100:1.66209421
36,(XP_023219960.1:0.0000020011,XP_023219961.1:0.0020478472)100:3.1437260
057)100:0.4126809419)92:0.8820508095,(((((((((((((((XP_015124116.1:0.429
9926245,XP_011300777.1:0.5188784632)100:1.3444833556,((XP_044016604.1:0.7
218442104,XP_044009238.1:0.9624938643)100:0.8887492211,XP_044019254.1:3.1
561257243)87:0.2702161162)87:0.2519763813,((XP_034949201.1:0.0000020151,X
P_034947667.1:0.0667613700)100:1.7655286651,XP_014295051.1:2.2811962143)1
00:0.6340654934)100:0.3850859322,((((((((((((XP_012163127.1:0.1186259280
,((XP_033354015.1:0.0000028404,XP_033354016.1:0.0000028404)100:0.02974524
89,((XP_033195433.1:0.0000028404,XP_033195434.1:0.0000028404)100:0.026402
5576,(XP_012249482.1:0.0000028404,XP_033180686.1:0.0000028404)100:0.02088
59218)96:0.0126551790)100:0.0502004062)95:0.0263983442,XP_043579971.1:0.0

720797435)100:0.4509860006,XP_043525578.1:0.5539183108)100:0.2183522503,(
(((XP_043799425.1:0.0244380725,(XP_006623214.1:0.0000028404,XP_031369828.
1:0.0000028404)100:0.0283804415)100:0.0243709282,((XP_028525746.1:0.00456
78161,(XP_016922533.1:0.0000028404,XP_028525745.1:0.0000028404)92:0.00000
21661)100:0.0379465715,XP_016773223.2:0.0575098065)99:0.0153395404)99:0.0
160872136,XP_012346658.2:0.0659266868)100:0.4215483749,XP_017766061.1:1.2
061623518)100:0.1159593834)100:0.1260558409,(XP_017890637.2:0.0000028404,
XP_017890636.2:0.0000026344)100:1.1848282984)100:0.1740482424,XP_01779899
3.1:1.0747458071)100:0.1115301332,(XP_012141619.1:0.5291352373,(XP_034173
241.1:0.0308430774,XP_029037837.1:0.0728559607)100:0.4326516386)100:0.425
1899408)100:0.1345801893,XP_043256536.1:0.9117606389)98:0.1228168581,(XP_
015432861.1:0.7735206318,(XP_031839433.1:0.7441008416,((XP_033323015.1:0.
0000598912,XP_033323016.1:0.0000028404)74:0.0018969977,XP_033323014.1:0.0
000022943)100:0.8828858525)100:0.1941252162)100:0.2964902470)100:0.489549
1951,(((XP_014597948.1:0.1179654097,(XP_043502988.1:0.0000028561,XP_04350
2989.1:0.0000028404)100:0.1129798232)100:0.0982515444,(XP_015183325.1:0.0
000028404,XP_015183335.1:0.0000028404)100:0.1737499909)100:0.5446067251,(
XP_043664522.1:0.4113440909,XP_035730111.1:0.3788374839)100:0.2407631332)
100:0.8344083973)95:0.0995985851,(((((((XP_029665438.1:0.0000027542,XP_02
9665441.1:0.0000028404)100:0.2643839166,(XP_025264753.1:0.0000021722,XP_0
25264754.1:0.0000028404)100:0.3256691782)100:0.1222598968,XP_029168478.1:
0.5299511273)100:0.2089122570,XP_012219118.1:0.6786274330)100:0.083832433
5,((((((((((XP_018043722.1:0.0000028404,XP_018043724.1:0.0000028404)100:
0.0069995317,XP_012055724.1:0.0094106598)100:0.1055593003,XP_011067950.1:
0.1368782923)100:0.0371163438,XP_018354963.1:0.1411029249)100:0.032928911
5,XP_018377133.1:0.1291909790)100:0.1000764449,XP_018303760.1:0.202080754
9)100:0.1239540557,XP_018402854.1:0.5622259095)100:0.2227781910,(XP_01169
3471.1:0.0000022288,XP_011693477.1:0.0000028404)100:0.5149663290)100:0.11
06664326,((XP_036140493.1:0.0000028404,XP_012543146.1:0.0000028404)100:0.
3461876146,XP_025991526.1:0.4631459159)100:0.1218493219)100:0.0565932801,
(XP_024871175.1:0.3111585001,XP_011871131.1:0.5356451628)100:0.0673426087
)100:0.1600114937,(XP_011632116.1:0.0000028404,XP_011632113.1:0.000002260
4)100:0.6138367951)100:0.2269647072)99:0.0716147116,(XP_011347403.1:0.668
9909175,XP_020280276.1:0.9843408630)99:0.1392701190)100:0.4986814640,(((X
P_032687794.1:0.0000022197,XP_032687848.1:0.0067474835)100:0.2120143890,X
P_025159612.1:0.3249853469)100:0.1102658042,XP_014473379.1:0.2889442321)1
00:0.7371012434)100:0.5143242106)100:0.2947760292,((((XP_033221780.1:0.02
43340319,(XP_033221778.1:0.0000028404,(XP_033221774.1:0.0000028404,XP_033
221777.1:0.0000028404)54:0.0000028404)98:0.0018583681)100:0.6053935422,(X
P_043469376.1:0.5040188551,(XP_043484526.1:0.0000028404,XP_043484524.1:0.
0000020514)100:0.9368014825)100:0.7779474341)100:0.7729077782,(XP_0122872
34.1:1.1958731158,(XP_043279609.1:0.0000028156,XP_043279610.1:0.000002840
4)100:1.7408396137)50:0.2087475655)47:0.1388701965,((((XP_024942926.1:0.0
209243324,XP_015599756.1:0.0000028404)63:0.0000026344,XP_015599757.1:0.00
00028404)59:0.0000023528,XP_024942927.1:0.0000028404)100:1.0926081320,(XP
_015511858.1:0.7104246073,XP_012265864.1:0.9116319357)100:1.0243136327)48
:0.1827482803)11:0.1202136966)100:0.3124208105,(((XP_031782799.1:0.945730
8784,XP_023246881.1:2.0470381894)100:0.2098794749,XP_011494459.1:1.866411
3106)100:0.2443034011,(XP_014238228.1:0.0000024002,XP_014238229.1:0.00000
28404)100:2.8613437452)100:0.4058749056)99:0.3103256426)100:1.2356756625,
(((((XP_019875477.1:1.9000670124,(((XP_018573469.1:0.8959447061,(XP_02302
9780.1:0.9805923281,XP_023020794.1:1.7705105964)96:0.1601498575)98:0.2285
999255,(XP_028146081.1:0.0000021895,XP_028146082.1:0.0213264651)100:1.671
7127582)100:0.1869003624,(XP_030753952.1:0.9186907830,(XP_019773736.1:0.0
000028404,(XP_019773735.1:0.0000028404,XP_019770526.1:0.0018513457)58:0.0
000025808)100:0.8781976891)100:0.9543936876)100:0.2149280322)100:0.253229
9551,NP_001107840.1:1.5860775286)100:0.5739635198,((((XP_017776930.1:0.00
80274589,XP_017780556.1:0.0000028404)100:0.0066527345,XP_017776931.1:0.00
00028404)100:2.6042662392,XP_022903592.1:2.0983754531)85:0.2626355856,(((
XP_031345654.1:0.0000021685,XP_031343315.1:0.0000028404)100:0.0204397752,
XP_031345881.1:0.0494819100)100:1.1285697937,XP_031333092.1:0.8278957405)

100:1.3199459210)81:0.1933429242)100:0.4472329079,XP_018331371.1:2.448833
1352)100:0.7702591672,((((((XP_026749163.1:0.7623299918,XP_013192187.1:0.
8070254419)75:0.1251244976,(XP_028170236.1:0.6471069566,XP_026319222.1:1.
1624005106)43:0.0938313823)91:0.1179495432,((((XP_022828203.1:0.179815490
7,XP_035450081.1:0.1736836930)100:0.3634266803,((XP_021197630.1:0.0000028
404,XP_021197628.1:0.0000028404)59:0.0000020514,XP_021197629.1:0.01116625
36)100:0.4226941857)98:0.1368171509,((XP_026733953.1:0.0000026342,XP_0267
44968.1:0.0000028404)100:0.2826509981,XP_026744965.1:0.0304792513)100:0.2
790091497)100:0.4874352435,((XP_028033553.1:0.0078330372,NP_001180543.1:0
.0089739692)100:0.8812132652,XP_037296279.1:0.8799451214)100:0.2339003950
)100:0.1862021404)90:0.0765197271,(XP_037969485.1:0.0021019900,XP_0379694
86.1:0.0000020278)100:1.3222247838)83:0.1052045596,(((XP_038213849.1:0.00
00028404,XP_038213845.1:0.0000026982)100:0.6577665951,XP_022115154.1:1.14
59542267)100:0.3407179816,(((((XP_023938601.1:0.0079319406,XP_023938579.1
:0.0000021116)100:0.4504075072,(XP_034829181.1:0.3630596172,XP_039755015.
1:0.3245998732)100:0.0877411986)100:0.3842673430,XP_026496410.1:0.7653896
268)100:0.1122342838,XP_032516970.1:1.0278509384)100:0.2693768263,XP_0419
80153.1:1.2229186089)79:0.1077701452)79:0.1212531194)61:0.1472419737,((XP
_013173437.1:0.2359957740,XP_014362498.1:0.1357718288)92:0.0864524974,XP_
013149103.1:0.2736738902)100:0.8514070782)100:2.1220046296)93:0.264672117
0)77:0.1072055222,(((XP_023722413.1:0.0000028404,XP_023722412.1:0.0000028
641)100:0.6736270455,(XP_021927093.1:0.0000028561,XP_021927095.1:0.000002
8404)100:0.7069995626)100:1.7716433667,(XP_026282585.1:1.1689411025,XP_03
4233796.1:1.3179892269)100:3.0922404640)61:0.3951102697)58:0.1849461963,(
(((XP_014251027.1:0.0000028404,XP_024083763.1:0.0000024002)100:2.04389321
26,(XP_014275310.1:0.0075200658,XP_014275311.1:0.0000022566)100:1.8028427
316)100:0.8179859563,((XP_039289133.1:0.0000022563,XP_039300577.1:0.00000
28404)100:0.0380353757,(XP_039299429.1:0.3829285970,XP_039299428.1:0.2662
084998)98:0.0754745513)100:2.9513214837)100:0.3263593263,(XP_018899828.1:
2.6699974602,(((XP_026819706.1:0.5548560206,XP_025190355.1:0.6322367509)1
00:0.4460090228,((((((XP_027844603.1:0.0000028404,(XP_027844607.1:0.00000
28404,XP_027844612.1:0.0000028404)96:0.0016558911)55:0.0000028404,XP_0278
44608.1:0.0000028404)49:0.0000026344,(XP_027844609.1:0.0000598912,XP_0278
44610.1:0.0000028404)87:0.0017086298)100:0.2426804808,XP_025207986.1:0.28
30418875)69:0.0353946952,((XP_026808825.1:0.0000732218,XP_026808826.1:0.0
000028404)95:0.0034359585,((XP_026808822.1:0.0000028404,((XP_026808823.1:
0.0000028404,XP_026808827.1:0.0000028404)100:0.0016517969,XP_026808828.1:
0.0000028404)62:0.0000028404)88:0.0000023961,XP_026808824.1:0.0000028404)
84:0.0000024002)100:0.2305498680)99:0.0670290282,(((((XP_015373396.1:0.00
00028404,XP_015373401.1:0.0000028404)88:0.0000028685,XP_015373402.1:0.000
0028404)51:0.0000028685,XP_015373404.1:0.0000028404)100:0.1932230099,(((X
P_016665099.1:0.0000028404,(XP_001945890.2:0.0000028404,XP_016665093.1:0.
0000028404)55:0.0000028404)83:0.0000026344,XP_003240109.1:0.0000028404)70
:0.0000022856,XP_016665103.1:0.0000028404)100:0.1609699224)69:0.035217681
7,((((XP_022180233.1:0.0000028404,XP_022180232.1:0.0000652136)90:0.003504
3271,XP_022180231.1:0.0000028404)59:0.0000028404,XP_022180227.1:0.0000028
404)62:0.0000028404,(XP_022180234.1:0.0000028404,XP_022180230.1:0.0000028
404)97:0.0016567780)100:0.2100338163)100:0.1237629497)100:0.1042433827)99
:0.2628064009,XP_025412382.1:0.5954021661)100:3.2688819696)100:0.38375934
35)68:0.3453784698)42:0.1887094171,(XP_026479503.1:2.7546836528,(XP_00242
5109.1:5.2415888992,(((((XP_035891817.1:0.6886411442,((XP_040234106.1:0.0
082049928,XP_320248.4:0.0073486701)100:0.0064845621,(XP_040163321.1:0.006
8413104,XP_041784077.1:0.0239726901)100:0.0042293426)100:0.4821169666)100
:0.5617733769,XP_035788113.1:1.1635362990)100:0.9479777237,((NP_001339903
.2:0.1885769993,XP_001652212.1:0.1895399125)100:0.5533446359,((XP_0381105
23.1:0.0000028404,XP_001855187.2:0.0000028404)100:0.0203493551,XP_0394471
99.1:0.0021792096)100:0.7298652708)100:0.7394697000)100:1.3497837430,((XP
_031635095.1:0.8190404378,XP_031624659.1:0.6851521930)100:1.6052934166,XP
_037029520.1:1.9293354628)100:0.3796411059)99:0.2791779829,(((((((((XP_0
17027359.1:0.1611776530,XP_020808394.1:0.1965530307)100:0.3738949911,((((
(((NP_523778.2:0.0000028404,NP_001286540.1:0.0016917919)100:0.2055907232,

((((XP_032572253.1:0.0000028404,XP_032572254.1:0.0000028404)78:0.00000239
61,XP_032572255.1:0.0161328215)100:0.0585664228,(XP_016028252.1:0.0000028
404,XP_039148315.1:0.0000028404)100:0.0500450028)91:0.0178355143,XP_03315
4392.1:0.0571547083)100:0.2105266583)100:0.3395563527,(XP_001974538.1:0.2
175718279,((XP_002092062.1:0.0241105736,XP_039483417.1:0.0188468988)100:0
.0429871472,XP_043646275.1:0.0401074294)100:0.0777122922)100:0.1191480419
)100:0.2152785388,XP_017078544.1:0.3535349771)98:0.0375047546,((XP_016946
969.1:0.1314419362,(XP_037714729.1:0.0372584308,XP_016929625.1:0.04739221
70)100:0.0349651776)100:0.1008855052,XP_016994522.1:0.1122618138)100:0.06
32958894)98:0.0600916881,(XP_017124526.1:0.1906079730,(XP_016976506.1:0.0
000021685,XP_016976507.1:0.0000028404)100:0.1708736624)100:0.1165014461)1
00:0.0406384796,XP_017049831.1:0.3351957451)100:0.1995082793)100:0.087563
4649,(XP_001960198.2:0.2636904688,XP_017094102.2:0.2704717534)100:0.44323
73149)100:0.1325088529,(((((((XP_017146129.2:0.0030640831,XP_033247174.1:
0.0036846311)100:0.0084516515,XP_033247154.1:0.0029060604)100:0.003463812
0,XP_033246402.1:0.0038054052)100:0.0819703750,(XP_001360634.4:0.01574346
32,XP_002016212.2:0.0063640522)100:0.0500850815)100:0.0105132062,(XP_0332
50700.1:0.0000028404,XP_033250699.1:0.0000022288)100:0.0947236106)100:0.0
118734005,(XP_033250702.1:0.0000028404,XP_033250701.1:0.0100971727)100:0.
0628401485)100:0.2538760341,(((XP_041450181.1:0.0051484568,XP_041450180.1
:0.0000028561)100:0.1765383970,(XP_022209697.2:0.0000028561,XP_022209698.
2:0.0034300450)100:0.2103461394)100:0.0592126373,(XP_034653529.1:0.113821
1212,XP_034120504.1:0.1574480097)100:0.2883815876)100:0.1020849733)100:0.
6463779938)100:0.1900759150,XP_002061021.2:0.9986168904)97:0.1673557990,(
((((XP_030567655.1:0.0351480565,XP_002049171.1:0.0336986610)100:0.2300926
803,(((XP_017856898.1:0.0444933071,XP_002006854.1:0.0178242894)100:0.0336
483646,XP_030245302.1:0.0762438648)100:0.1273524356,XP_023178200.2:0.2483
006834)100:0.2500994478)100:0.0923038106,XP_001985724.2:0.5891186026)100:
0.0789328762,(XP_034479318.1:0.3048740340,XP_034109801.1:0.5739973418)100
:0.1418164945)100:0.1205979031,XP_017838829.1:0.6792204897)100:0.20699296
86)97:0.2374533870,(XP_030375295.1:0.0050020578,XP_030375294.1:0.00000273
81)100:0.6513535187)100:1.2767786938,((((XP_005187138.1:0.7388619561,XP_0
13098531.1:0.7279292739)100:0.3825295274,(XP_023297775.1:0.1191070389,(XP
_037825226.1:0.0000023961,XP_037826510.1:0.0169883386)100:0.1129934957)10
0:0.6084780143)100:0.3025090390,XP_037887702.1:1.0985620061)100:0.4048709
953,((XP_011180498.1:0.2802257232,((((XP_018797270.1:0.0000028404,XP_0187
97244.1:0.0000028404)100:0.1257522273,(XP_039953901.1:0.0000028404,XP_039
953903.1:0.0000028404)100:0.0465714373)94:0.0153863559,(XP_029405432.1:0.
0000028404,XP_011201131.1:0.0000028404)100:0.0499576902)100:0.1200245446,
(XP_036230897.1:0.0000028404,XP_036230896.1:0.0000028404)100:0.1534185053
)100:0.1958857429)100:0.3159385991,(XP_004529883.1:0.3205031928,((XP_0363
33291.1:0.0036845407,((XP_017476504.1:0.0310203624,XP_017476506.1:0.01268
53165)100:0.0538997620,(XP_017476503.1:0.0466003569,XP_017476505.1:0.0284
852783)100:0.0310172047)100:0.0560546520)100:0.0080478170,XP_017478671.1:
0.0277932315)100:0.3932349825)100:0.1416830168)100:0.7957510479)100:0.283
9541006)89:0.2483780395,XP_037956391.1:1.7820307387)100:1.3112853188,(XP_
037914929.1:0.0000028404,XP_037914928.1:0.0000023961)100:2.0820866126)100
:0.5923363247)100:1.1668786017)68:0.3255375262)53:0.3147312678)89:0.23124
10748,(XP_034253907.1:1.1792604983,(XP_026289781.1:0.0169374442,XP_026292
484.1:0.0194922907)100:1.9250279527)100:4.5274432450)98:0.3761645093,(XP_
002430037.1:4.3150822552,((XP_020906600.1:0.0000028641,XP_028516578.1:0.0
000028404)100:2.5150268713,(XP_013391116.1:2.2645221630,(XP_024141959.1:0
.0000028404,XP_024141962.1:0.0020194135)100:2.3513160057)100:0.6305650021
)100:3.4455950854)95:1.4419558095)62:0.0574012797,XP_026686328.1:4.593018
5548)73:0.2772157628,(((XP_021960857.1:0.2583969559,XP_021945451.1:0.2283
722758)100:3.6531029700,XP_035704405.1:3.9893908350)100:0.7607149386,(XP_
027198147.1:2.1724931461,XP_027197670.1:2.2551307031)100:3.5524363196)81:
0.3166124546)66:0.2721885574,(((XP_032796029.1:0.8927163576,XP_032789032.
1:0.7617513846)100:1.6338992828,XP_032779841.1:2.3107907117)100:1.1856197
025,XP_031637122.1:6.9622837310)84:0.4863215431)82:0.1883903406,(((((XP_0
42237352.1:1.5107723556,((XP_027207363.1:0.1382549042,XP_037801760.1:0.14

93067446)100:0.0925259384,((XP_042887206.1:0.0000027708,XP_042856442.1:0.
0322263002)100:0.2437033616,XP_027238253.1:0.9094431197)100:0.1018061186)
100:1.4025573221)100:2.4214039639,XP_018013061.1:3.6108723963)100:0.89140
45917,(XP_002108754.1:2.9492132672,XP_002107959.1:4.1356909169)100:1.4504
118555)98:0.3326174647,(XP_040578741.1:3.3594351654,(XP_023325914.1:0.000
0022708,XP_023325915.1:0.0047439266)100:4.9257762350)100:1.5405576847)93:
0.3241396786,((((XP_022708785.1:0.0021497464,XP_022643620.1:0.0000028404)
100:0.9870668545,XP_028968970.1:0.8626449384)100:1.8669909189,((((XP_0226
65039.1:0.0000028404,XP_022665038.1:0.0000020452)100:0.0294372949,XP_0226
65037.1:0.0000021237)100:0.0108208888,XP_022694219.1:0.0109468899)100:2.2
660718038,(XP_003746827.1:0.9067879817,XP_018497627.1:3.0710403834)100:0.
9266699282)100:1.3651270939)100:1.7620054477,((((XP_022645213.1:0.0000028
404,XP_022645209.1:0.0000028404)100:0.0035042348,(XP_022708791.1:0.000002
8404,XP_022708787.1:0.0000028404)78:0.0000020536)100:1.4919871547,(XP_028
968733.1:0.8655051901,XP_028968740.1:0.8412138096)100:1.0620740856)100:0.
6144819182,XP_028966508.1:2.6155220263)100:1.6940280855)100:1.4574682959)
54:0.2147341590)50:0.1187764672,(XP_037032546.1:2.4647379624,XP_015784164
.1:3.6965850042)100:2.0000020516)37:0.1145648816,(((((((((((XP_027042533
.1:0.2690619268,(((XP_027042516.1:0.0424318302,XP_027042518.1:0.102916704
4)100:0.0264372535,(XP_027042510.1:0.0070747763,XP_027042511.1:0.02910848
82)100:0.0786697497)99:0.0541309980,XP_027042512.1:0.2505470349)59:0.0173
153780)98:0.0105175712,XP_027042517.1:0.2426163612)100:0.0786023454,(XP_0
22788048.1:0.0582000576,XP_022788047.1:2.0395024347)100:0.1652553791)100:
0.1619640805,XP_022788046.1:0.8014061170)100:0.5109199912,XP_020618184.1:
0.6139197659)100:0.2095805237,(XP_020615803.1:0.7442609620,((XP_029179993
.1:0.0639057865,(XP_015776860.1:0.0233811299,XP_029197459.1:0.0610909612)
100:0.1205419862)92:0.1371236618,XP_015776861.1:0.3191005887)100:0.914601
1674)100:0.2806079853)100:1.1829225735,((XP_020894938.1:1.3990135149,XP_0
31558371.1:1.0266285163)100:0.7171926302,XP_032231423.1:1.8624518803)100:
0.7558976720)100:0.4619669532,(XP_028398771.1:0.5168336855,XP_028398776.1
:0.6084402977)100:3.0572174842)100:0.8786296309,(XP_012561318.1:0.3052605
256,XP_012556556.1:0.1349763980)100:4.2160984931)100:0.2133878567,(XP_019
850113.1:2.5553863150,((XP_003384628.3:0.2109621125,XP_003391904.2:0.0692
433194)100:0.7492608489,XP_019854673.1:3.1509355044)100:2.1587493955)100:
1.6467592801)59:0.2273288161,((((((XP_020618826.1:0.9117819029,(XP_027058
140.1:0.3264041674,XP_022805684.1:0.3683258629)100:0.7531074824)100:0.474
1001418,((XP_029197160.1:0.0000023192,XP_029197161.1:0.0080946730)100:0.0
582027342,XP_015758828.1:0.0252075483)100:1.5629970971)100:1.1692959417,(
(XP_020910298.1:2.3024136912,(XP_031563858.1:0.0048417281,XP_031563857.1:
0.0000021912)100:1.6218849747)100:1.1026205505,(XP_032232625.1:0.00000284
04,XP_032232624.1:0.0000025808)100:2.9357827520)100:0.8545822240)100:0.91
93292748,XP_009176136.1:9.3910276491)100:0.6293770767,XP_028398652.1:4.85
46902733)100:1.0292561499,XP_002107798.1:5.7622932592)53:0.2668256435)90:
0.2010843344,XP_021195705.1:8.6646660815)65:0.1392982317)81:0.1611616955)
100:0.3167750424,(((((XP_003137813.1:0.1345644374,XP_042933082.1:0.104202
7838)100:1.0787724180,(((NP_498761.2:0.3800312030,(XP_003104996.1:0.27795
48548,XP_002643058.1:0.3143194570)100:0.2141172384)100:0.8671013291,XP_01
3293536.1:0.8758507582)100:0.2095095662,XP_013293534.1:0.8069096793)100:0
.4517859962)100:1.1030488628,XP_024503581.1:4.4516135458)100:0.6270583394
,(XP_003375890.1:1.9216240036,XP_004622446.1:1.6555126136)100:1.679937689
2)82:0.5468813594,((((XP_018644375.1:0.0790629247,XP_035589668.1:0.071696
1542)100:1.0060949068,XP_009167121.1:1.0430647896)100:0.7163638266,XP_024
350050.1:2.5910912435)100:1.4261677368,((XP_003377020.1:0.0000022563,XP_0
03366828.1:0.0101334157)100:0.0218564832,XP_003368069.1:0.0135744014)100:
4.0717946965)82:1.1229706966)93:0.2479377246)92:0.2600492608)63:0.2107241
536,XP_014663886.1:1.9130458812)93:0.1951048522)100:0.5670750545)100:0.23
79129282)100:0.3492790792,((XP_039260611.1:0.0010911646,XP_039260610.1:0.
0000027017)100:1.1840052702,XP_009860329.1:1.1241463325)100:1.7934300790)
100:0.5711258893)100:0.2147659365)94:0.0821785041,(((XP_006632316.1:0.128
1496612,(((XP_035281664.1:0.1150177088,XP_036397855.1:0.0828165603)100:0.
0466108828,(((((XP_030637558.1:0.0054162726,XP_030637551.1:0.0013857307)1

00:0.1515895529,(((XP_022529006.1:0.1137156223,(XP_036449832.1:0.01250008
72,XP_017566772.2:0.0322390893)100:0.0332836489)100:0.0255302186,(((XP_02
6782396.2:0.0471146212,(XP_017332678.1:0.0000028404,XP_017332675.1:0.0000
023961)100:0.0993069011)100:0.0129850845,XP_027015698.1:0.0749762767)100:
0.1946623591,(XP_026880051.2:0.0033762882,XP_035388780.1:0.0372796766)100
:0.2353119784)100:0.0461484386)100:0.0806695329,(((XP_043118449.1:0.02130
30042,((XP_018975754.2:0.0214479336,XP_026141406.1:0.0421683502)100:0.008
4932590,((XP_016316028.1:0.0184764505,XP_016125401.1:0.0480751483)100:0.0
041054434,XP_016421924.1:0.0360336339)100:0.0158051488)100:0.0039489155)1
00:0.0062849284,((XP_016090306.1:0.0060971095,(XP_016379216.1:0.009672066
6,XP_016319449.1:0.0118073057)100:0.0014292311)100:0.0159034414,(XP_02611
5952.1:0.0198675011,XP_042629761.1:0.0193676889)100:0.0205759882)100:0.01
36602612)100:0.0053270448,(XP_039526851.1:0.0874377494,NP_001154925.1:0.0
816866107)99:0.0096362379)100:0.1451329502)100:0.0459294426)100:0.0403531
760,XP_028846066.1:0.2378845484)99:0.0372211645,(XP_041927934.1:0.1333436
640,(XP_031435633.1:0.0000028404,XP_012692254.2:0.0025926163)100:0.138441
2418)100:0.2299584471)99:0.0509307817,(((XP_029900029.1:0.0732975766,((((
(((((XP_041809395.1:0.0226336101,(XP_011616905.1:0.0000027542,XP_02968640
4.1:0.0000028404)100:0.3215301093)91:0.0007259559,((XP_036981113.1:0.0132
006824,XP_030247920.1:0.0044027438)100:0.0535614415,(XP_019120723.1:0.000
0028404,XP_019120722.1:0.0000028404)100:0.0485620309)100:0.0132270789)93:
0.0054941225,XP_035529995.1:0.0853105189)99:0.0070214239,(((XP_044021715.
1:0.0000028404,XP_044021717.1:0.0000028404)100:0.0000021661,(XP_044021718
.1:0.0000020244,XP_044021719.1:0.0000028404)100:0.0238779945)100:0.034652
9882,XP_038583942.1:0.0762225884)100:0.0186187461)93:0.0031851068,((XP_03
3499564.1:0.0332316169,XP_042356160.1:0.0411930680)100:0.0055935001,((((X
P_028422209.1:0.0065366080,XP_039642591.1:0.0059925922)100:0.0111152594,X
P_031177300.1:0.0118304038)100:0.0085857452,(XP_032355697.1:0.0125736289,
XP_034713543.1:0.0280333832)100:0.0300665894)100:0.0584773230,(((((((XP_0
33934194.1:0.0266013947,XP_033991085.1:0.0101187099)59:0.0000020820,(XP_0
10775252.1:0.0000014837,XP_010785563.1:0.0121431394)42:0.0531359638)73:0.
0000020514,XP_010789126.1:0.0000028404)64:0.0014163891,XP_034053930.1:0.0
094630640)99:0.1519519409,XP_029316245.1:0.0848270446)98:0.0320251303,((X
P_034418941.1:0.0912968816,(XP_040055515.1:0.0715984196,XP_037342492.1:0.
0349244829)100:0.0949328717)100:0.0061794062,XP_031731727.1:0.0573863108)
100:0.0511459602)98:0.0089351391,XP_037652363.1:0.0543875052)99:0.0134492
494)99:0.0073313993)99:0.0137560957)93:0.0076106472,((((XP_018553217.1:0.
0322945964,(((XP_022606466.1:0.0000028404,XP_023250497.1:0.0040534596)100
:0.0282120728,XP_029349978.1:0.0679179028)100:0.0198707238,((XP_039993998
.1:0.0000021661,XP_039994000.1:0.0082017427)100:0.0356122081,XP_040916213
.1:0.0434119054)89:0.0036932385)89:0.0014809933)74:0.0034995530,(((XP_035
465360.1:0.0000027542,XP_035465363.1:0.0632216054)100:0.1237412041,(XP_01
9958629.1:0.0505414501,((XP_034432990.1:0.0000028404,XP_034432991.1:0.001
4100731)100:0.0013566705,XP_035024293.1:0.0013277577)100:0.0344406887)100
:0.1090206788)100:0.0128643705,(XP_043903163.1:0.0855793807,XP_008316059.
1:0.2388337551)100:0.0793778368)100:0.0224179167)100:0.0133747708,((XP_02
0454890.1:0.0833032960,(XP_026159256.1:0.0000028404,XP_026159506.1:0.0000
028404)100:0.1175675391)100:0.0157401489,((XP_026211510.1:0.0000028404,XP
_026211512.1:0.0000028404)100:0.0719957179,(XP_028994466.1:0.0000028404,X
P_028994467.1:0.0000028404)100:0.1477407103)100:0.0458706390)74:0.0067359
541)74:0.0038138286,(((((XP_008294508.1:0.0395450848,(XP_022077099.1:0.01
73197616,XP_023145500.1:0.0108154895)100:0.0229197752)100:0.0503929832,XP
_028250506.1:0.1346082070)74:0.0084481491,(((XP_031596231.2:0.0026877620,
XP_019204140.1:0.0027024950)100:0.0065694413,((((XP_013770625.1:0.0040742
017,(XP_014195969.1:0.0000028404,XP_042084874.1:0.0000023961)100:0.002696
9433)75:0.0000023961,XP_039905062.1:0.0013472884)100:0.0013475159,XP_0127
78180.2:0.0013479872)100:0.0074063809,XP_035761756.1:0.0493251721)100:0.0
124982263)100:0.0628751885,XP_030580845.1:0.0662615949)100:0.0671168503)6
7:0.0061735726,(XP_029972007.1:0.0944341386,(XP_028293377.1:0.0000028404,
XP_028293380.1:0.0000028404)100:0.2999705313)100:0.0450367598)52:0.011333
3916,((XP_041827819.1:0.1516545636,(XP_024128907.1:0.0352077103,XP_011488

451.1:0.0474514719)100:0.1649372878)83:0.0193994339,((((((XP_014865745.1:
0.0061860275,(XP_007553167.1:0.0017499559,XP_014903755.1:0.0040906108)100
:0.0058618715)100:0.0130235600,XP_008398343.1:0.0190354377)100:0.00631982
49,(XP_043999110.1:0.0176323776,((XP_032402940.1:0.0000028404,XP_03240293
8.1:0.0000028561)100:0.0058722672,(XP_023208571.1:0.0058576257,XP_0278562
05.1:0.0058519186)100:0.0014637335)100:0.0078175790)100:0.0087338929)100:
0.0170845887,(XP_038163139.1:0.0029190960,XP_015250180.1:0.0058100567)100
:0.0424940513)99:0.0082288683,XP_036007041.1:0.0754826884)100:0.085849154
6,((XP_017287169.1:0.0389036978,(XP_013867302.1:0.0487027914,XP_037551072
.1:0.0914989143)100:0.0177097836)100:0.0378918875,XP_015830712.1:0.206569
8038)100:0.0442730976)100:0.0698852169)100:0.0840231933)100:0.0229955250)
92:0.0057074284)91:0.0120587316,(XP_042245084.1:0.0456231526,(XP_02049692
7.1:0.0662185361,(XP_034530472.1:0.0601702692,(XP_041669419.1:0.000002396
1,XP_041669420.1:0.0016329973)100:0.0771909679)100:0.0181812653)100:0.062
5349554)95:0.0094900265)96:0.0073606800,(XP_019725553.1:0.1009177902,((XP
_037096546.1:0.0000028404,XP_037096550.1:0.0000028404)100:0.0000022856,XP
_037096551.1:0.0080188992)100:0.1830812237)100:0.2653932693)92:0.00995336
51,((XP_029983073.1:0.0622858300,(XP_020786351.1:0.0158593192,XP_03384384
3.1:0.0260898053)100:0.2204923111)98:0.0295635103,XP_034050915.1:0.267887
4664)62:0.0140171346)100:0.0877462668)100:0.0533748926,XP_030211677.1:0.4
190384201)100:0.1106378753,(XP_010877055.2:0.1190580541,(((((XP_038865742
.1:0.0123512788,XP_023849823.1:0.1141158894)100:0.0199190185,(XP_02142956
4.2:0.0308105255,(XP_042186542.1:0.0529329981,XP_031684100.1:0.0502413367
)100:0.0204767088)100:0.0330762864)100:0.0100688936,((XP_029585515.1:0.00
00028404,XP_029585518.1:0.0012473219)100:0.0083188911,(XP_014050520.1:0.0
000028404,XP_014050548.1:0.0012345245)100:0.0152661141)100:0.0135212723)1
00:0.0422737102,XP_041704139.1:0.0309779905)100:0.0640101245,((((((XP_014
066777.1:0.0062385122,XP_029568424.1:0.0200037545)100:0.0178623918,((XP_0
38822916.1:0.0075708441,XP_023842732.1:0.0107130938)100:0.0011637534,XP_0
23842070.1:0.1996029337)98:0.0282899034)93:0.0141054695,((XP_036819037.1:
0.0068273176,XP_029531886.1:0.0122300610)100:0.0052930877,XP_031643756.1:
0.0085514606)93:0.0023262589)91:0.0187855060,XP_035615861.1:0.0580538438)
95:0.0214995475,XP_042182185.1:0.0239340570)100:0.0572552754,XP_041710110
.1:0.0458956758)100:0.0502944516)100:0.0235523921)100:0.1731411027)100:0.
1052389075)100:0.0996160707)97:0.0241898430,(XP_018583251.1:0.1609860173,
XP_023649635.1:0.2655753201)100:0.1473307769)99:0.1225860355)99:0.0514356
833,(((XP_033893800.2:0.0027357182,XP_034771689.1:0.0000028404)100:0.0082
443229,(XP_033891399.2:0.0027196721,XP_034783126.1:0.0027236876)100:0.002
7550637)100:0.0027905307,(XP_041132494.1:0.0406568135,XP_041121615.1:0.04
14406428)100:0.0179193805)100:0.1622310418)84:0.0378813275,(XP_028677929.
1:0.0095521668,XP_039629332.1:0.0156591992)100:0.2604481841)99:0.20158590
58)93:0.0393270677)100:0.1154609800,(((XP_018085949.1:0.0430487941,(XP_04
1429979.1:0.0068317756,NP_001163918.1:0.0882552306)100:0.0739389940)100:0
.0169687772,(XP_031746094.1:0.0000028404,NP_001123390.2:0.0000028561)100:
0.0508540177)100:0.1604181625,(XP_040268805.1:0.1796029819,(XP_018414903.
1:0.0790884790,XP_040189568.1:0.2080349975)100:0.1121659353)100:0.0860772
721)100:0.2137294167)100:0.0330783313,((XP_029442355.1:0.0000025199,(XP_0
29453260.1:0.0410592256,XP_029453259.1:0.3627519022)71:0.1054837587)100:0
.0804841537,(XP_030069596.1:0.0405097170,XP_033809161.1:0.0404633237)100:
0.0565681052)100:0.1919244453)100:0.0597758025)98:0.0360275199,(XP_038600
376.1:0.0129210012,XP_028926941.1:0.0163699519)100:0.0825159077)100:0.016
2279133,(((XP_001367933.3:0.0000028404,(XP_016281007.1:0.0000028404,XP_01
6281008.1:0.0264119523)99:0.0000028714)100:0.0169338885,(((XP_020860797.1
:0.0074253439,XP_027712333.1:0.0046538388)100:0.0041906582,XP_036608144.1
:0.0154606631)100:0.0018290979,XP_003756622.2:0.0098294490)100:0.00501595
42)100:0.0027084890,XP_043845226.1:0.0280091875)100:0.0502128315)100:0.11
13588577,((((XP_037687884.1:0.0000028404,XP_037687883.1:0.0000021661)77:0
.0000021661,XP_037687885.1:0.0013303027)100:0.0277389596,XP_004467704.2:0
.0511427875)100:0.0208448085,(XP_003408901.1:0.0412180836,(((XP_023587534
.1:0.0000021116,XP_023587527.1:0.0000028404)99:0.0000021702,XP_023587533.
1:0.0167502759)100:0.0646662855,(XP_007942714.1:0.1276827513,(XP_00683960

8.1:0.1060204693,(XP_004699154.1:0.1115424751,XP_012859411.1:0.6883633513
)100:0.1676806258)100:0.0409673126)85:0.0156458784)70:0.0042593430)99:0.0
103835139)96:0.0038410922)96:0.0097663010,((((((((XP_023986905.1:0.00000
28404,(XP_028352166.1:0.0000028404,XP_036697316.1:0.0228291222)76:0.01855
22413)75:0.0000028404,XP_028352167.1:0.0319290268)76:0.0077741600,(((XP_0
04262420.1:0.0000028404,(XP_030739323.1:0.0012896998,(((XP_024614143.1:0.
0000028404,(XP_024614177.1:0.0000028404,(((XP_032477154.1:0.0017889359,XP
_024614190.1:0.0000028404)50:0.0000020472,XP_024614183.1:0.0000028404)56:
0.0037989489,XP_032477153.1:0.0732065924)84:0.0067075052)51:0.0000026194)
99:0.0013215878,(XP_029093042.1:0.0025889144,XP_022431885.1:0.0025928891)
99:0.0012969410)100:0.0106370972,XP_007447384.1:0.0143079520)86:0.0000026
194)60:0.0000026194)51:0.0000026194,XP_026976260.1:0.0012894947)59:0.0000
026194,XP_019773990.1:0.0012895713)100:0.0173518920)76:0.0014059803,(XP_0
36697313.1:0.0038636221,XP_007178959.1:0.0064446728)76:0.0089639805)100:0
.0116450257,(((((XP_010815510.1:0.0000028404,((XP_005894318.2:0.000002840
4,XP_010849706.1:0.0025893849)100:0.0012953370,NP_976235.1:0.0142904340)1
00:0.0000028404)100:0.0000028404,XP_019838934.1:0.0080149140)99:0.0012858
176,XP_025127082.1:0.0012954855)99:0.0025681262,((XP_027813165.1:0.007805
6312,XP_017921670.1:0.0052210764)100:0.0051554080,XP_040113030.1:0.010394
3041)100:0.0039025156)99:0.0038773788,((XP_043777251.1:0.0000028404,(XP_0
43345316.1:0.0051718810,XP_020754798.1:0.0103773191)98:0.0012807811)95:0.
0000028404,((XP_043777258.1:0.0000028404,XP_043345324.1:0.0065695108)79:0
.0000024002,(XP_043777256.1:0.0000028404,XP_043345322.1:0.0065341211)100:
0.0128683599)95:0.0110097095)99:0.0038403585)100:0.0191109766)100:0.00727
80431,((XP_013844676.2:0.0000028404,NP_001184123.1:0.0013359789)95:0.0000
028404,XP_020953825.1:0.0425465100)100:0.1086705147)100:0.0071190657,(XP_
006213971.2:0.0022896246,((XP_010967049.1:0.0000028404,(XP_031310118.1:0.
0012972859,((XP_032338463.1:0.0000028404,XP_031310124.1:0.0013353016)100:
0.0078938923,XP_010967051.1:0.0318184602)100:0.0173853271)77:0.0000028641
)95:0.0000028641,XP_032338462.1:0.0080457253)100:0.0015964504)100:0.03323
72538)99:0.0142547525,(((((((XP_021535502.1:0.0000028404,XP_021535504.1:0
.0000028404)99:0.0000028404,((XP_030894556.1:0.0000028404,XP_030894558.1:
0.0000028404)100:0.0012871199,(XP_034848623.1:0.0000028404,XP_034848628.1
:0.0000028404)100:0.0064362161)100:0.0012828631)100:0.0025643594,(XP_0322
61752.1:0.0000028404,XP_035951507.1:0.0000028404)100:0.0012811300)100:0.0
038768709,((XP_004394570.1:0.0000028404,XP_012415759.1:0.0399758140)100:0
.0038614400,((((XP_027425245.1:0.0000028404,XP_027425251.1:0.0000028404)9
9:0.0000028404,XP_027425252.1:0.0066229554)100:0.0012864257,XP_027954746.
1:0.0025674881)88:0.0000028404,XP_025725198.1:0.0038578293)100:0.00256172
92)100:0.0143526787)100:0.0074522724,((((XP_026371150.1:0.0000028404,XP_0
40497415.1:0.0000028404)100:0.0090706477,XP_002920522.2:0.0051725413)100:
0.0154136708,(((XP_030175748.1:0.0012935792,(((((XP_043412882.1:0.0000028
404,XP_043412883.1:0.0000028404)99:0.0000028561,(XP_043412884.1:0.0000028
404,XP_043412885.1:0.0107838812)72:0.0000020299)72:0.0038906633,(XP_02311
1766.1:0.0000028404,XP_023111768.1:0.0000028404)100:0.0025873286)50:0.000
0598912,XP_008707588.1:0.0000028404)62:0.0012352294,(((XP_042800139.1:0.0
000028404,(((XP_019317729.1:0.0000028404,XP_019317731.1:0.0000028404)100:
0.0000971686,XP_042800141.1:0.0000028404)100:0.0024977111,(XP_042846401.1
:0.0000028404,(XP_007094984.1:0.0000028404,XP_007094986.1:0.0000028404)10
0:0.0025952582)100:0.0025943756)68:0.0000028404)66:0.0000028404,XP_042800
140.1:0.0000028404)78:0.0000028561,XP_042800142.1:0.0000028404)99:0.00779
79133)75:0.0000028404)74:0.0000028404,(((XP_040334094.1:0.0000028404,XP_0
40334096.1:0.0000028404)100:0.0038884463,XP_025774555.1:0.0038869897)77:0
.0000028404,(XP_026922342.1:0.0000028404,XP_026922344.1:0.0000028404)100:
0.0012936936)96:0.0025872675)98:0.0245946362,(XP_039110487.1:0.0344491278
,(XP_029806065.1:0.0000028404,XP_029806068.1:0.0000028404)100:0.087486375
3)100:0.0420747146)99:0.0344886396)98:0.0041269129,((XP_022360821.1:0.005
2170944,((XP_032693358.1:0.0000028404,XP_032693361.1:0.0000028404)94:0.00
00028404,XP_032693363.1:0.0027001172)100:0.0065740764)100:0.0026254171,((
(XP_032201058.1:0.0000028404,XP_032201062.1:0.0000028404)89:0.0000028404,
XP_032201063.1:0.0027078116)100:0.0026323685,XP_004754801.2:0.0091921591)

100:0.0013185679)100:0.0197572552)95:0.0017544290)97:0.0038963379,(XP_025
838383.1:0.0000028404,XP_022278061.1:0.0025673378)100:0.0164616115)100:0.
0099890370,((((XP_011377899.1:0.0000028404,XP_006925624.2:0.0012920619)10
0:0.0000028404,(XP_039740066.1:0.0000028404,(XP_039740072.1:0.0000024002,
XP_039740069.1:0.0128885753)97:0.0110279204)95:0.0025684931)95:0.00774269
29,(XP_036073892.1:0.0000028404,(XP_036073897.1:0.0000028404,((XP_0360738
96.1:0.0000024002,XP_036073895.1:0.0128284613)86:0.0000022513,XP_03269378
1.1:0.2096976048)83:0.0000022490)95:0.0110900886)93:0.0090124736)98:0.041
1369198,(((XP_019498029.1:0.0000028404,(XP_019498033.1:0.0018744758,XP_01
9498028.1:0.0349830741)100:0.0135127865)100:0.0193938915,(XP_032963251.1:
0.0000028404,XP_032963260.1:0.0000028404)100:0.0373048844)100:0.030789133
5,((((XP_036109149.1:0.0000028404,XP_036109190.1:0.0000028404)100:0.04423
99746,(XP_016056495.1:0.0000028404,XP_016056497.1:0.0186970714)100:0.0389
669020)100:0.0085295337,((((XP_006106222.2:0.0000028404,(XP_023602742.1:0
.0000028404,XP_023602741.1:0.0000028404)100:0.0029058066)100:0.0043876443
,(((XP_005871220.2:0.0000028404,XP_014399140.1:0.0000028404)38:0.00000284
04,(XP_014399143.1:0.0000027632,XP_014399144.1:0.0000028404)89:0.01265804
92)63:0.0000028404,XP_014399142.1:0.0052559482)96:0.0012775534)97:0.00000
25840,(((XP_036173840.1:0.0000028404,(XP_036173916.1:0.0000028404,XP_0361
73905.1:0.0000024461)100:0.0128601321)70:0.0000028404,XP_036173895.1:0.00
53344594)100:0.0173297787,(XP_015422178.1:0.0000028561,(XP_015422180.1:0.
0000028404,XP_015422179.1:0.0000023883)100:0.0102544556)100:0.0270180919)
96:0.0050233713)99:0.0248729712,(((XP_008157769.1:0.0000028404,XP_0279922
72.1:0.0000028404)99:0.0000028404,(XP_027992269.1:0.0000028404,(XP_027992
273.1:0.0000028404,XP_027992274.1:0.0054351949)61:0.0000028404)84:0.00000
28404)100:0.0455634157,((((XP_036281682.1:0.0000028404,XP_036281687.1:0.0
000028404)55:0.0000028404,XP_036281686.1:0.0000028404)69:0.0000028404,XP_
036281688.1:0.0112812189)89:0.0000028641,(XP_036281690.1:0.0000023742,XP_
036281691.1:0.0000028404)98:0.0136378864)100:0.0629235161)100:0.025604447
8)100:0.0466973222)100:0.0107801883,(XP_024423167.1:0.0203950599,(((XP_03
6986019.1:0.0000028404,XP_036986022.1:0.0000028404)100:0.0256571842,(XP_0
36887563.1:0.0000028404,XP_036887568.1:0.0000028404)100:0.0688543399)100:
0.0137314065,(XP_028361775.1:0.0000028404,(XP_035869567.1:0.0000028404,XP
_035869582.1:0.0000028404)100:0.0013111025)100:0.0766239607)100:0.0142787
902)100:0.0350943777)100:0.0139375694)83:0.0051809438)94:0.0099568529)82:
0.0032681503)94:0.0022339380,(((XP_004434298.2:0.0000028641,(XP_014646003
.1:0.0000028404,XP_014646002.1:0.0000598912)100:0.0013755122)100:0.040675
2400,((XP_014698956.1:0.0000028404,((XP_023484100.1:0.0000028404,XP_00853
3215.1:0.0104748010)97:0.0000028404,XP_023484103.1:0.0308076652)98:0.0025
912711)98:0.0363075147,XP_012790688.1:0.3993540896)98:0.0193603097)80:0.0
093367506,((XP_036737883.1:0.0073664105,XP_036847418.1:0.0136830003)100:0
.0559976379,((XP_037359865.1:0.0131397408,XP_004681647.1:0.0516810657)100
:0.0749284378,(XP_006903693.1:0.0908507050,XP_016046281.1:0.3476676115)93
:0.0620744986)77:0.0105841299)72:0.0081988607)58:0.0028830099)76:0.012791
2245)80:0.0064849223)78:0.0047979796,(XP_006165433.2:0.0842756684,XP_0215
68565.1:0.1824160884)96:0.0230234840)96:0.0050735881,XP_008568746.1:0.019
2917567)97:0.0022617246,((((XP_012610821.1:0.0000028404,XP_012610830.1:0.
0000028404)96:0.0000028404,XP_012610829.1:0.0183302398)100:0.0099434634,X
P_012500961.1:0.0105992196)100:0.0109218814,XP_003787065.1:0.0226161527)9
7:0.0035171625)100:0.0238140553,(((XP_012313714.1:0.0025420706,(XP_017383
680.1:0.0000028404,XP_032143398.1:0.0025419848)100:0.0038127931)87:0.0000
028404,XP_039324639.1:0.0012692933)97:0.0000028404,(XP_002754306.1:0.0000
028404,XP_035117285.1:0.0091652323)100:0.0063479727)97:0.0063590979)100:0
.0014999181,(((XP_023061325.1:0.0000022203,(XP_011806909.1:0.0000028404,X
P_011806908.1:0.0037670283)99:0.0050175424)99:0.0025103234,(XP_033047344.
1:0.0012714022,(XP_030786338.1:0.0025412248,(XP_017713423.1:0.0000028404,
XP_017713425.1:0.0120013689)100:0.0043590819)96:0.0000028641)100:0.001450
2866)76:0.0000028641,((XP_003902281.1:0.0000028404,(XP_025247615.1:0.0000
028404,(XP_025247614.1:0.0000028404,XP_011847073.1:0.0050234235)80:0.0037
632858)58:0.0012524032)52:0.0012525000,(((XP_011939230.1:0.0025435966,(((
XP_023061330.1:0.0027316019,XP_033047348.1:0.0027284831)100:0.0027277821,

XP_011939238.1:0.0027251529)85:0.0000028404,XP_021797222.1:0.0013623516)1
00:0.0110016794)46:0.0000028404,XP_007985916.1:0.0051044797)15:0.00000284
04,((NP_001244801.1:0.0000028404,XP_011715396.1:0.0012707207)100:0.001276
8675,XP_011847074.1:0.0025482787)23:0.0000028404)50:0.0000028404)98:0.002
5067097)99:0.0000021685)100:0.0046497319)100:0.0022988352)98:0.0025588028
)48:0.0000028404,((((((XP_001154369.1:0.0012702476,XP_034793957.1:0.00254
33971)100:0.0000631431,NP_001382620.1:0.0000028404)32:0.0000598912,((((NP
_001382615.1:0.0000028404,NP_001382616.1:0.0000028404)60:0.0000028404,XP_
016782173.1:0.0013664020)75:0.0000028404,XP_034793964.1:0.0027370834)83:0
.0000028404,XP_018865320.1:0.0013660158)99:0.0120051636)17:0.0000598912,N
P_001382626.1:0.0000028404)26:0.0000598912,XP_011534907.1:0.0000028404)96
:0.0010246615,(NP_001382614.1:0.0051871802,((NP_001382621.1:0.0000028404,
NP_001382625.1:0.0114887354)100:0.0100595079,(NP_001182502.1:0.0000028404
,XP_016782174.1:0.0025907879)100:0.0000024906)99:0.0309769327)90:0.000002
2375)44:0.0000028404);
